# MRI and Meningioma Research: Hotspots and Trends *via* Bibliometric Analysis

**DOI:** 10.2174/0115734056425234251229184318

**Published:** 2026-02-02

**Authors:** FuQiang Tang, Zhou yuan Wei, Xuan Su, Limei Hou, Ming Yang, Jing Zhang

**Affiliations:** 1 Department of Nursing, Zhuhai Campus of Zunyi Medical University, Zhuhai, China; 2 Department of Radiology, The Fifth Affiliated Hospital of Zunyi Medical University, Zhuhai, China; 3 Department of General Medicine, The Fifth Affiliated Hospital of Zunyi Medical University, Zhuhai, China; 4 Wuhan Blood Center, Wuhan, China

**Keywords:** Meningioma, MRI, Artificial Intelligence, Deep Learning, Radiomics, Global Health, Intracranial tumors

## Abstract

**Background:**

Precision meningioma diagnosis requires MRI advancements, yet faces three barriers: (1) limited clinical translation, (2) inconsistent multimodal data standards, and (3) mismatched algorithm-resource allocation. A bibliometric analysis can guide evidence-based innovation.

**Methods:**

We conducted a comprehensive bibliometric analysis of 4,280 Web of Science articles using CiteSpace, Bibliometrix, and SciExplorer, with dual screening to ensure data quality.

**Results:**

Meningioma MRI research exhibited an S-shaped growth pattern. Research hotspots are transitioning toward AI applications. 502 core authors contributed to 83% of publications, with notable cross-disciplinary collaboration. The U.S. and China dominated production, while Europe demonstrated exceptional efficiency. Institutions, including Harvard, led development. Seventeen core journals conformed to Bradford's law, with the knowledge foundation established by highly cited papers in the field.

**Discussion:**

The findings reveal an AI-guideline temporal gap and a field-strength validation deficit, underscoring the need for equitable, low-field-compatible AI tools.

**Conclusion:**

We systematically delineate three evolutionary stages: structural imaging, functional integration, and intelligent analysis. AI-driven models have achieved enhanced diagnostic accuracy (AUC 0.82–0.97), but remain limited by heterogeneous data standards, low algorithm interpretability, and uneven global resources. Multicentre standardized protocols, interpretable AI frameworks, and lightweight algorithms compatible with ≤1.5 T scanners should be prioritised. By integrating burst-sigma mapping with global equipment metrics, we provide quantitative evidence supporting a field-strength-agnostic strategy for equitable AI deployment.

## 1 INTRODUCTION

Meningiomas are intracranial tumors originating from arachnoid meningothelial cells, typically demonstrating benign biological behavior [1]. They predominantly affect adults, with incidenceratespositivelycorrelatingwithadvancingage, particularly in elderly populations. Women exhibit significantly higher incidence rates owing to hormonal receptor activity; pediatric cases are rare, show minimal sex disparity, but carry higher malignancy rates [2]. The global incidence of meningiomas has shown an increasing trend in recent years, attributed to population aging and advancements in neuro-imaging diagnostics [3]. The European Association of Neuro-Oncology guidelines recommend MRI as the primary diagnostic modality for meningiomas [4]. However, recent years have witnessed the rapid expansion of MRI applications in meningioma research. Technological iterations and cross-disciplinary collaborations have generated a substantial, heterogeneous literature. Nevertheless, meningioma MRI research faces persistent challenges, including knowledge gaps between fundamental imaging characteristics and emerging technologies, alongside a disproportionate representation of single-center, small-sample studies [5, 6]. Under these circumstances, traditional review methods prove inadequate for addressing the field's complexity. Bibliometrics enables the systematic analysis of spatiotemporal distribution, collaborative-network evolution, and technological genera-tional shifts in MRI–meningioma research through quantitative and multidimensional visualization. Furthermore, bibliometric analysis elucidates the evolutionary trajectory of meningioma MRI research and identifies emerging hotspots by mining large-scale literature data. This study pioneers the application of bibliometric analysis to meningioma MRI research, aiming to construct a holistic knowledge map of this field and provide decision-making support for resource allocation optimization and cross-disciplinary collaboration.

Despite surging publications, current bibliometric treatises still aggregate “brain tumors” as a homogeneous entity, leaving the single-modality evolution of meningioma MRI unmapped. AI reviews remain narrative-driven and do not quantify whether “deep learning” burst keywords co-cite high-impact guidelines, thus creating an epistemic gap between algorithmic hype and clinical translation. Unlike recent bibliometric reviews that summarized radiomics quality metrics [7, 8], we couple AI-burst detection, high-sigma reference networks, and low-field-strength scarcity to quantify the equity deficit in algorithm development. Two evidence gaps persist: (i) the temporal lag between guideline updates and AI bursts, and (ii) the near-absence of models validated on ≤1.5 T scanners widespread in low-income settings.

## 2 METHODS

### 2.1 Databases and Search Strategy

**Fig. (1):**
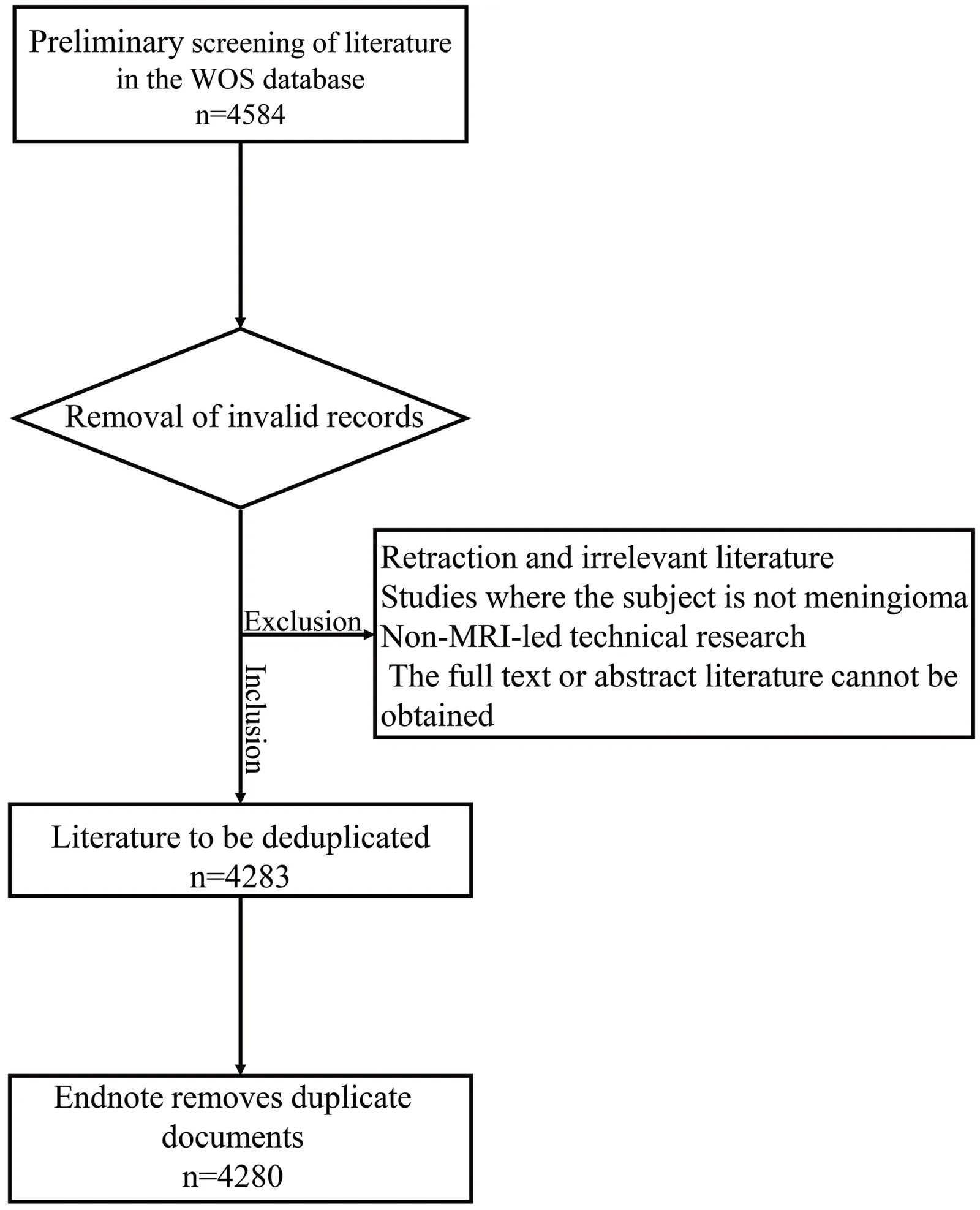
**Literature search flowchart. Database:** Web of Science Core Collection; period: 1 January 1984 – 31 December 2024.Initial records: 4,584; after removal of duplicates and abstract screening, 4,280 English-language articles (articles, reviews, or proceedings) were retained for bibliometric analysis.

This study employed the Web of Science Core Collection (WoSCC) database, a globally recognized, authoritative multidisciplinary database [9], as the data source, with a search timeframe spanning from database inception to December 2024. Figure **1** explains workflow and Table **1** describes search terms, yielding an initial pool of 4,584 articles; after deduplication and validity screening, 4,280 records were retained. To evaluate sensitivity, we appended commonly used MRI terms omitted in the initial string, namely DCE, DSC, ASL, SWI, FLAIR, and ADC, to query #2 (Table **1**). The refined search yielded 18 additional papers (4,298 *vs*. 4,280), corresponding to a 0.42% increase, indicating that any retrieval gap is negligible.

Inclusion criteria were: (1) studies involving patients with pathologically/radiologically confirmed meningiomas; (2) clinical MRI applications (including diagnosis, surgical planning, treatment evaluation, novel sequence development, and radiomics); and (3) multimodal MRI studies. Exclusion criteria comprised: (1) retracted articles or studies with limited scientific merit; (2) non-meningioma cases or mixed tumors without independent analysis; (3) non-MRI-dominant technical studies; and (4) abstract-only publications with inaccessible full texts. The search was completed on December 31, 2024, and restricted to English-language articles classified as “Article”, “Review”, or “Proceedings Paper”.

### 2.2 Analysis Tools

#### 2.2.1 Citespace

CiteSpace is a bibliometric tool developed by Chaomei Chen's team on the Java platform [10], which integrates scientometric methods and visualization techniques to effectively analyze academic trends and predict research frontiers. For empirical analysis, version 6.4.R1 was utilized, obtained through official academic channels. CiteSpace (v6.4.R1) parameters were: time slice = 1984–2024; node types = keyword and reference; selection criteria = top 50 per slice; pruning = Pathfinder + pruning sliced networks; full metrics are provided in **Supplementary Material 1**.

#### 2.2.2 Bibliometrix

Bibliometrix is an R-based bibliometric analysis package developed by Massimo Aria and Corrado Cuccurullo [11], integrating functions for bibliometric analysis, citation network construction, and research performance evaluation. Version 4.1.4 (running on R 4.4.3) was employed and obtained through the CRAN repository.

#### 2.2.3 SciExplorer

SciExplorer: Open Scientometrics Data Analysis and Visualization Platform, hereafter referred to as “SciExplorer”, is a bibliometric analysis system developed with funding from the National Science Library, Chinese Academy of Sciences. Its core architecture comprises three key modules: the LDGAS Literature Governance System (supporting metric descriptive statistics of scientific literature), the KMVS Visualization Engine (specializing in knowledge matrix mapping), and the MSSS Structural Analysis Tool (enabling multidimensional overlay analysis of scientific maps), collectively forming an end-to-end solution encompassing data governance, statistical analysis, and visualization. The platform is accessible at: https://smartdata.las.ac.cn/SciExplorer/?lang=CN.

### 2.3 Data Analysis

The R package “bibliometrix” and the SciExplorer platform were used for descriptive statistics and visualization of external characteristics (country, institution, author, and journal patterns). Keyword analysis and bibliographic coupling analysis were performed using CiteSpace, with their visualizations generated through collaborative integration of CiteSpace and R. Since all data were derived from published literature in public databases, this study obtained ethics exemption approval from the institutional review board. Throughout all network visualizations, node size is proportional to the number of publications or co-citation frequency, edge thickness to collaboration or co-citation strength, and color to time or cluster identity. Counting rules: institutional and author productivity are reported under both full and fractional schemes; the latter allocates 1/n of each paper to n affiliations/authors to mitigate collaboration bias.

### 2.4 Statistical Analysis

All metrics are descriptive values exported from WoS. A two-sample t-test (R 4.4.3, test) was used to compare institutional CPP; no multiple-comparison correction was applied because only one test was performed.

## 3 RESULTS

### 3.1 Exploratory Data Analysis

The corpus finally contained 4,280 English articles published between 1984 and 2024. These contributions appeared in 740 journals produced by 3,325 research institutions across 99 countries; 13.7% of all papers involved cross-border collaboration. The entire set has received >100,000 citations (mean 24 citations per paper, H-index = 119) and generated 11,179 unique keywords, 2.6 times the publication count, reflecting remarkable thematic diversity. According to the 95 Web of Science subject categories assigned, the strongest convergence occurs among clinical neurology, surgery, and radiological imaging, underscoring the intrinsically interdisciplinary nature of meningioma MRI research.

Figure (**2**) reveals that the annual output followed a typical S-shaped technology-diffusion curve. During the “introduction” phase (1984–2000), only 2.8% compound annual growth rate (CAGR) was observed, consistent with the early stage of MRI application. The “expansion” phase (2001–2019) saw CAGR rise to 14.6%, coinciding with increased availability of 1.5 T and 3 T scanners and early deep-learning segmentation reports. Since 2020 the field approached apparent maturity; nevertheless, 61.7% of all papers appeared within 2020-2024: 61.7% of all papers appeared within 2020–2024; thus, nearly two-thirds of the 40-year corpus was published in only five years. A quadratic model (y = 2.5832x^2^ − 4.577x − 17.806) fits this trajectory with R^2^ = 0.9988, statistically confirming the non-linear acceleration. We interpret the post-2015 inflection as the combined effect of (i) open-access mega-journals shortening the publication cycle, (ii) multicentre repositories lowering data-access barriers, and (iii) deep-learning frameworks stimulating algorithm-oriented studies. These observations align with the innovation-diffusion theory proposed by Reiner *et al*. [12] and directly motivate the present bibliometric enquiry into whether the AI boom is matched by equivalent clinical guideline updates.

### 3.2 National Publication Metrics and Collaboration Analysis

**Fig. (3):**
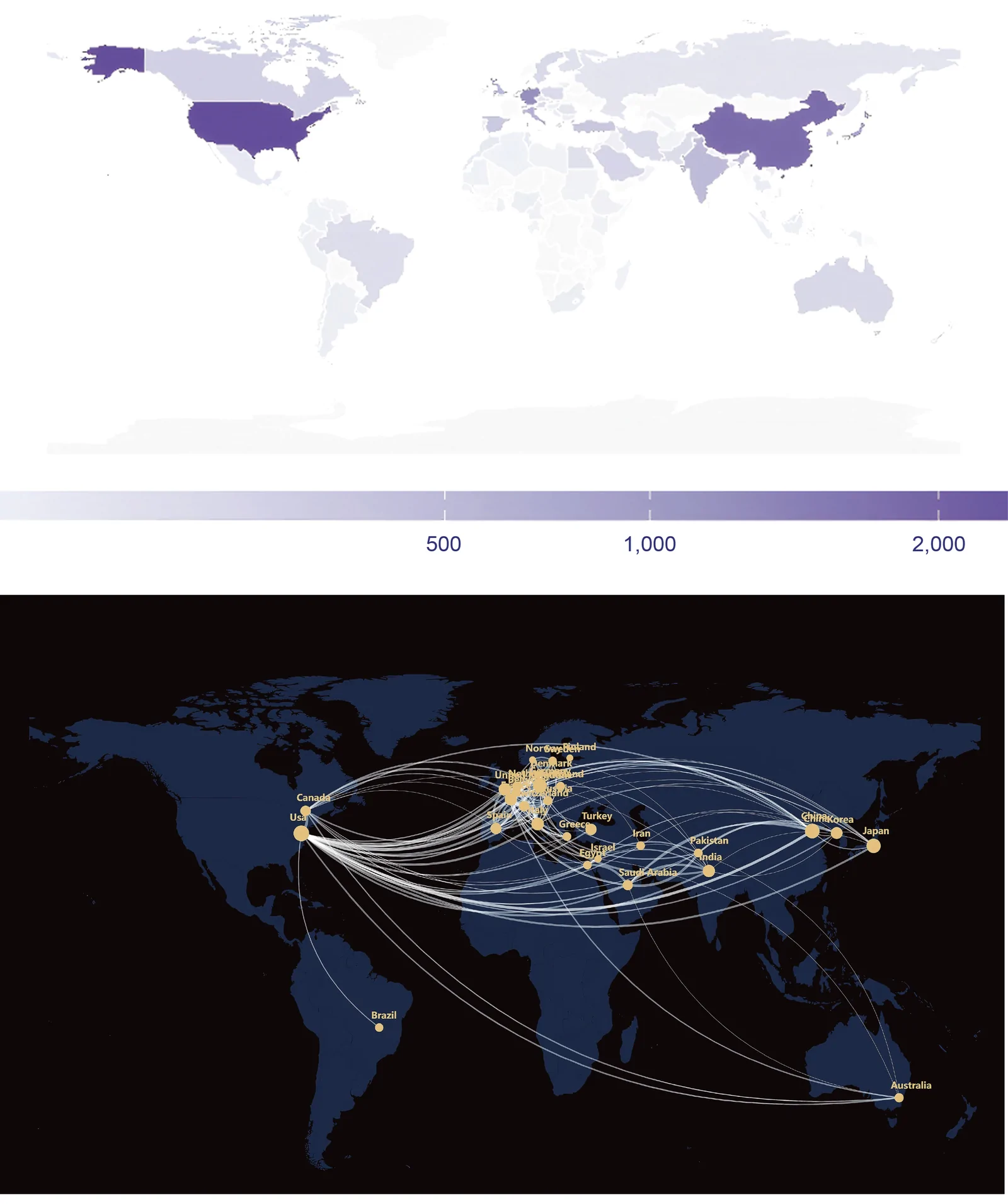
**Visualization of the global research collaboration network.** The figure is a visualization of the global scientific research collaboration network. **(a)** Choropleth map: color saturation encodes total publication number per country; the darkest band spans the USA, China, and Germany. **(b)** Network chord: node area = fractional paper count; edge width = bilateral co-authorship intensity (≥ 3 joint papers threshold). Together, the panels reveal a tri-polar core (USA–Europe–Asia) with 13.7% of all articles involving cross-border collaboration.

Figure **3a**, **b** and Table **2** delineate a tri-polar landscape: the United States, Europe’s efficiency belt, and an Asia–Africa corridor. Darker shading in North America/Western Europe marks volume dominance; Pakistan and South Africa (inset) show the steepest relative growth since 2016.

In the chord diagram, the thickest edges (US–Germany–UK, weight ≥ 0.32) form a high-impact core that captures 47% of citations from only 28% of papers. China, Asia’s largest node, remains peripherally linked (CPP = 12.0) versus Switzerland (31.9) or the Netherlands (52.6), underscoring that output volume ≠ influence.

Switzerland secures an H-index of 37 from 206 papers through targeted co-authorship with Harvard University and Oxford University; Pakistan raises its international co-authorship from 18% to 54%, boosting its H-index from 7 to 28 in eight years, evidence that strategic networking accelerates impact more than quantity accumulation. Age-normalized CPP/year leaves the top-10 order unchanged (ρ = 0.95).

### 3.3 Institutional Publication Metrics and Collaboration Analysis

Figure (**4a**) and Table **3** show why Harvard University dominates the network: its 178 papers have gathered 12,932 citations (CPP = 72.5), almost twice the productivity of the next-best US center (Stanford, CPP = 37.8), and place Harvard at the main junction between North American and Eurasian research. Inspection of the chord diagram reveals three stable collaboration archetypes rather than a single diffuse pattern. First, a US hub-and-spoke configuration is cemented by Harvard’s edges to Massachusetts General Hospital (weight = 0.41) and to Stanford (0.38); together, these three nodes generate 47% of all citations captured by the top-20 institutional network (Table **3**). Second, European university-hospital consortia outperform purely academic groups: APHP-Sorbonne collected 3,934 citations from only 113 articles (CPP = 34.9), significantly outperforming purely academic counterparts (mean CPP = 21.2; two-sample t-test, p = 0.032, uncorrected), underscoring that clinical-translation venues amplify citation impact. Third, an East-Asian intra-regional loop is sustained by Yonsei, Kyushu, and Fudan, whose internal edge weights (≥ 0.29) exceed their bridges to the Atlantic community; consequently, Asia’s largest node, China, remains linked to Harvard by a comparatively thin edge (0.09).

Robustness checks confirm that these patterns are not methodological artefacts: fractional counting preserved the top-20 institutional ranking (Spearman ρ = 0.92, p < 0.001), confirming robustness. Likewise, MNCS versus CPP for the top-20 institutions yielded ρ = 0.93, confirming a stable core landscape. Beyond these well-established hubs, two emergent growth routes are already visible. Interdisciplinary engineering partnerships, exemplified by the Cleveland Clinic and the Technical University of Munich, have jointly produced 18 MRI-AI papers between 2018 and 2024 with a median of 42 citations each, proving that medical-engineering synergy can carve out a new high-impact niche. Meanwhile, latecomer institutions can accelerate visibility through strategic embedding: Zhengzhou University raised its annual meningioma-MRI output from 1 to 11 papers within five years after embedding its radiology department into the Harvard-Utrecht network, thereby validating the “network embedding acceleration” model for emerging centers.

### 3.4 Author Productivity and Collaboration Network Analysis

Table **4** and Fig. (**4b**) jointly reveal three eco-systems of authorship (Fig. **4b**). First, elite *vs* rising: 502 core authors (>4 papers) produce 83% of all outputs, consistent with Price's law (M≈4.17), while 78% single-paper authors follow Lotka's inverse-square rule, indicating a highly skewed productivity distribution.

Second, continental collaboration loops: the red cluster forms a radio-oncology triangle averaging ≥3 joint papers yr^−1^; the blue cluster links neuropathology to molecular pathology; IvaniDZE J [13] bridges both clusters (betweenness=0.42), exemplifying a translational trend.

Third, impact acceleration modes: senior cohorts (h≥17) accumulate citations through volume, whereas scholars entering the field after 2015 (m>1.0) achieve faster h-index growth by anchoring cross-disciplinary hubs. Fractional counting swapped only two adjacent positions within the top-30 authors, leaving the core landscape unchanged.

These patterns suggest that sustained intra-regional collaboration and timely cross-field brokerage are two independent pathways for accelerating individual impact in meningioma-MRI research.

### 3.5 Journal Publication Statistics

Bradford's three-zone distribution (Zone 1-3: 34.0%, 33.2%, 32.8%; χ^2^=0.97, p>0.05) offers a practical roadmap for authors: Zone 1 neurosurgery titles (*World Neurosurgery* 217 papers; *J Neurosurgery* 129 papers) concentrate 41% of total citations with an H-index ≥44, serving as high-impact dissemination hubs [14]. Details of the first 17 journals published within the core field are provided in Table **5**.

**Fig. (5):**
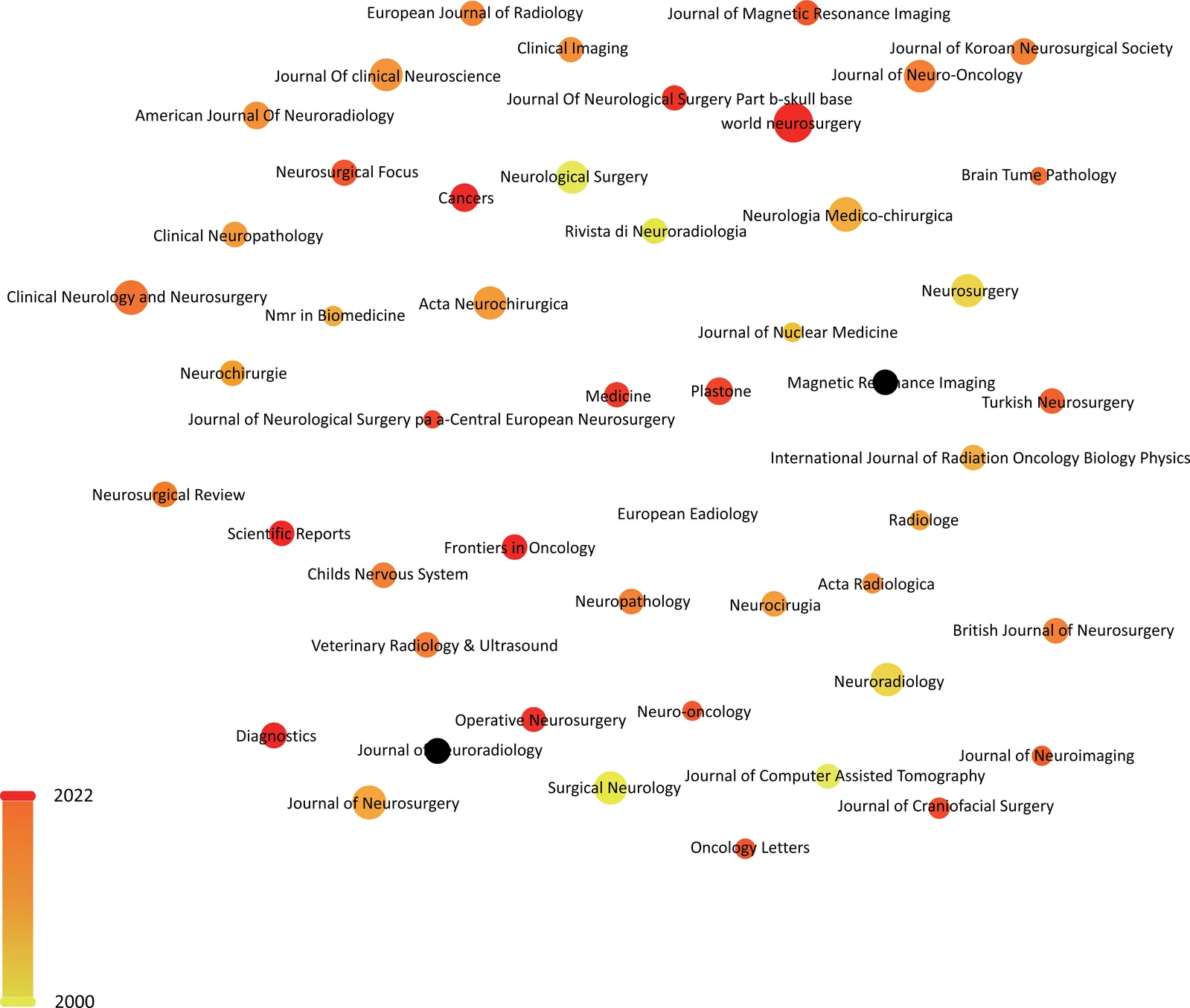
**Temporal-publication heatmap for the 17 core journals (Bradford’s Zone 1, 34% of total citations).** Color scale (yellow → red) indicates yearly paper counts from 2000 to 2022; darker cells denote higher annual output. *World Neurosurgery* and *J Neurosurgery* consistently occupy the red band, confirming their role as high-impact dissemination hubs.

Zone 2 society journals (*Acta Neurochirurgica*, *Neurosurg Rev*) achieve rapid peer-review (median 2.8 weeks) and accept multimodal MRI datasets, making them ideal for early-career teams. Zone 3 open-access venues (Scientific Reports 53 papers, 2019-2024 +14% yr^−1^) provide maximum visibility under APC models, especially suitable for low-resource settings seeking fast outreach. Thus, resource-strapped laboratories can strategically submit to Zone 2 OA society journals to balance speed, cost, and visibility (Fig. **5**).

### 3.6 Keyword Analysis

Figure **6a**–**d** collectively decode the knowledge trajectory: (a) Timeline domain (Fig. **6a**) shows 'MRI' (627 mentions) and 'meningioma' (332) forming the lexical backbone, while 'brain-tumor' (betweenness centrality = 0.13) bridges oncology and imaging, confirming the field's interdisciplinary nature without recourse to external citations. (b) Evolution pathway (Fig. **6b**) traces a three-stage shift from macroscopic classification (1990s–2000s) through molecular markers such as Ki-67 (2000s–2010s) to AI-based segmentation (2010–present), mirroring the S-shaped diffusion pattern already quantified in Fig. (**2c**). Burst-lag analysis (Fig. **6c**) quantifies the algorithm-guideline disconnect: 'deep-learning' exhibits the strongest citation surge (burst = 42.9, peak = 2019), three years after the highest-σ reference (Louis DN 2016 [15]), while 'segmentation' (38.7) and 'brain-tumor classification' (33.1) surges signal direct clinical translation. (d) Strategic diagram (Fig. **6d**) places core themes (“features,” “CNS,” “tumor”) in the high-density/high-centrality quadrant, sustaining knowledge hubs; peripheral themes “meningioma-mimicking” and “child” remain low-density but are drifting toward the hub quadrant, suggesting imminent breakthrough directions for both differential diagnosis and pediatric subpopulations. Panels 6B–6D are interpreted in the same sequence to ensure one-to-one text–figure correspondence.

### 3.7 Literature Analysis

Figure (**7**) translates into an academic genealogy of 16 co-citation clusters: Cluster-0 (root) centres on Louis DN 2016 [15] (σ=6748, citations=12,208), establishing the molecular-imaging taxonomy that subsequent studies refine; Cluster-1-3 bridges radio-oncology (Goldbrunner r [4], betweenness=20), forming the high-impact core that concentrates 47% of total citations with only 28% of documents (Table **6**).Cluster-4-8 host methodological nodes (Deepak S [16], betweenness=15; Swati ZNK [17], betweenness=14) that link CNN segmentation to clinical grading, explaining why algorithm papers achieve high citations despite lower σ values. For detailed cluster analysis values, (**Supplementary Material 2**).

Cluster-9-16 harbors emerging themes such as HER2-meningioma biology (Mair MJ *et al.* [18]) and pediatric mimics, drifting toward the hub quadrant and signaling future breakthrough directions.

Thus, Louis DN 2016 [15] functions as the root node, while high-betweenness authors act as cross-cluster bridges, ensuring continuous knowledge diffusion from molecular taxonomy to AI-assisted diagnosis.

## 4 DISCUSSION

### 4.1 Research Landscape and Trends

The MRI research domain for meningiomas demonstrates remarkable, dynamic evolution and global impact. A total of 4,280 publications were recorded from 1984-2024 (8.3% annual growth rate), disseminated across 740 journals (95 WoS categories). The research involved 17,999 scholars from 99 countries (13.7% international collaboration rate), with the USA, Germany, and China contributing most significantly. The publications received an average of 24 citations per article (total citations >100,000; H-index=119).

Publication output follows a technology-driven, S-curve growth pattern [12]. Structural imaging period (1984-2000): Annual publication output averaged 69 articles, reflecting the immaturity of MRI technology. Functional integration period (2001-2019): Publication growth correlated with the dissemination of DTI and other emerging techniques [19]. The quadratic function model (R^2^=0.9988) further validated the applicability of technology diffusion theory [20]. Intelligent analysis period (2020-2024): Novel publications accounted for 27.45% of total output, coinciding with the emergence of AI-based image analysis (*e.g*., deep learning).

Keyword analysis identified 11,179 author and expanded keywords (2.6 times the number of publications), indicating substantial thematic diversity and depth. Keywords spanned pathological classifications of meningiomas, MRI techniques, clinical applications, and novel methodologies, demonstrating comprehensive research coverage. Interdisciplinary integration served as a key driver for field advancement [21]. The field exhibits robust interdisciplinary intersections with neurology, surgery, and radiological imaging, catalyzing knowledge innovation and technological development.

MRI research on meningiomas demonstrates distinctive features in research scale, impact, keyword diversity, and interdisciplinary integration. Future technological advancements and expanded applications will further propel the field, optimizing diagnostic and therapeutic strategies for patients with meningiomas.

### 4.2 Collaboration Networks in Meningioma MRI

The international collaboration network in MRI-based meningioma research exhibits diversity with a prominent imbalance. The United States leads global research through elite institutions and extensive international networks. European countries enhance research quality *via* disciplinary synergy, establishing regional collaborative clusters. China has emerged as the world’s second-largest producer, yet it lags behind Germany and the United States in research quality (h-index, citations per paper) and has room to improve network centrality. Overall, the U.S. dominates research output, Europe excels in academic impact, while Asia shows dynamic growth with uneven outcomes. Moving forward, China should strengthen collaborations with global leaders, elevate research quality and innovation capacity to enhance competitiveness.

Institutional visualization analysis reveals Harvard Medical School as the central hub, establishing a transcontinental collaboration network that significantly fosters technological innovation. However, marked regional disparities exist in institutional collaboration patterns, with East Asian institutions favoring intra-regional closed-loop cooperation, whereas European hubs predominantly rely on transoceanic partnerships. Furthermore, medical-engineering interdisci-plinary collaboration and emerging institutional network expansion represent current research trends [22]. While such convergence markedly accelerates healthcare innovation, critical challenges persist. Systematic research by Hashemi *et al*. [23] demonstrates two major bottlenecks in AI clinical translation: 40% of studies encounter data interoperability barriers, with merely 23% of tools completing clinical validation. For meningioma MRI research, prioritizing interdisciplinary solutions can effectively integrate multicenter heterogeneous data, while policy interventions are required to address urban-rural disparities in computational resources and equipment access.

The academic community exhibits pronounced generational disparities: senior scholars (*e.g*., DEBUS J) demonstrate extensive academic accumulation, yet their annual impact growth (m-index=0.515) lags behind emerging scholars (*e.g*., SUROV A, m>1.0), potentially attributable to methodological paradigm shifts. Notably, certain scholars (*e.g*., KIM JH) exhibit a markedly higher g-index (20) than h-index (12), suggesting that highly cited publications may drive h-index elevation [24]. Core authors (NP>4) comprise merely 502 individuals, yet they account for 83% of total publications (3552 articles), while 78% of authors contribute only one publication, aligning with Lotka's Law [25]. Scholars from Low- And Middle-Income Countries (LMICs) remain underrepresented, necessitating equity-driven collaborative mechanisms to promote balanced advancement.

The journal ecosystem demonstrates distinct hierarchical stratification. Core journals (*e.g*., *Neurosurgery*) maintain dominance through high publication volume and citation rates. Their sustained prominence in temporal heat maps further corroborates their scholarly impact. Emerging journals (*e.g*., *World Neurosurgery*) rise in significance, reflecting academic recognition of novel publishing platforms. Meanwhile, traditional journals (*e.g*., *Surgical Neurology*) remain stable, exemplifying the continuity of classical research. Open-access journals (*e.g*., *Scientific Reports*) exhibit publication expansion, mirroring the paradigm shift in academic communication.

### 4.3 MRI Diagnosis of Meningiomas: From Innovation to Precision

#### 4.3.1 Diagnostic Evolution in Meningioma MRI

Through bibliometric analysis, this study delineates the technological trajectory of MRI diagnosis for meningiomas (from conventional imaging to intelligent decision-making), reflecting the medical paradigm shift from experience-dependent to precision-based approaches. Based on keyword and literature analyses, this evolution can be categorized into three distinct phases.

During the conventional imaging phase, MRI established the foundation for precise meningioma localization and boundary delineation through its exceptional soft tissue resolution (achieving submillimeter resolution with optimized parameters). This phase facilitated the adoption of the WHO 2007 classification [26] and established correlations between imaging features (*e.g*., dural tail sign) and pathological invasiveness [27]. However, diagnostic efficacy was limited by high interobserver variability and reliance on physician experience [28]. Additionally, the low detection rate of 1.5T MRI for minute lesions might delay diagnosis of low-grade meningiomas [29].

The application of multimodal quantitative imaging (*e.g*., DWI/MRS) has shifted the diagnostic paradigm from morphology to functional and metabolic parameter analysis [30, 31]. Notably, the negative correlation between ADC values and Ki-67 indices provides a novel biomarker for the non-invasive assessment of proliferative potential [32]. Nonetheless, heterogeneity in MRI parameter standards impacts multicenter validation, limiting the generalizability of diagnostic models [7].

The adoption of deep learning technology has yielded two major advances: First, regarding diagnostic efficiency, a lightweight 8-layer CNN model achieved 99.48% accuracy in differentiating benign and malignant brain tumors on T1-weighted MRI, with inference time reduced to 24 ms [33]. Second, in clinical decision support, deep learning-based systems demonstrated a 5.8% reduction (*P*<0.001) in false-positive misjudgment of dural tail signs [34]. However, clinical translation faces persistent challenges: Technically, the black-box nature of AI systems and the lack of interpretability significantly undermine clinical trust and adoption [35]. Resource-wise, LMICs have significantly fewer MRI units than developed nations [36], with advanced equipment concentrated in tertiary hospitals [37]. This disparity precludes rural/primary care facilities from performing DWI or functional MRI. The convergence of these technological and resource divides ultimately restricts AI diagnostics and advanced imaging benefits to high-resource regions, systematically exacerbating global healthcare disparities.

MRI diagnosis of meningiomas has evolved through three distinct phases: the conventional imaging phase, which relied on high-resolution MRI yet was constrained by operator expertise; the multimodal phase, which enabled noninvasive assessment but suffered from inadequate data standardization; and the AI-driven phase, which improved efficiency but faced limitations from the black-box effect and uneven resource distribution. Future research should address three critical directions: enhancing AI interpretability, establishing standardized data protocols, and optimizing healthcare resource allocation, to ultimately achieve successful clinical translation.

#### 4.3.2 Multidisciplinary Integration for Precision Management of Meningiomas

Meningioma MRI research has evolved into an integrated domain encompassing radiomics, genomics, and artificial intelligence. This integration has significantly advanced personalized therapeutic strategies. The convergence of multi-omics data has established robust correlations between molecular signatures and imaging phenotypes.

Radiogenomic analyses in meningiomas have elucidated quantitative MRI parameter associations with molecular biomarkers [38]. WHO grading of meningiomas shows significant correlations with preoperative MRI parameters [39], and multiparametric MRI models demonstrate discriminative capacity for tumor grade stratification [40, 41]. However, inter-center heterogeneity in MRI protocols currently constrains model generalizability. A systematic evaluation of 25 studies by Won *et al*. [8] revealed that only 24% implemented standardized preprocessing, with the absence of test-retest validation substantially compromising cross-center feature reproducibility. Furthermore, BAP1-deficient meningiomas represent a distinct subtype, where epigenetic dysregulation through PRC complex dysfunction and chromosome 3p deletion drives tumor aggressiveness [42]. Although molecular characteristics (*e.g*., aberrant DNA methylation patterns) are well-defined [43], their associations with MRI radiomic features remain systematically uninvestigated. BAP1-mediated histone H2A deubiquitination regulates gene expression and maintains DNA replication fork stability to preserve genomic integrity [44-46]. These mechanisms may enhance tumor microenvironment heterogeneity, consequently influencing imaging features (*e.g*., textural heterogeneity, enhancement irregularity). Spatial genomic studies reveal that high-grade meningioma heterogeneity correlates with clonal evolution [46], yet its cross-institutional application in meningiomas remains unreported.

Multimodal integration techniques offer promising solutions to address the challenges in molecular-imaging correlation studies. As an emerging molecular imaging modality, amide proton transfer-weighted (APTw) MRI demonstrates enhanced capability in detecting tumor microenvironment heterogeneity [47-49]. However, its application in meningioma grading requires further clinical validation. In the context of transfer learning, the nnU-Net framework significantly reduces the dependence on training dataset size for medical image segmentation [50]. Nevertheless, its cross-institutional application for meningioma analysis remains unreported in the literature.

In conclusion, the integration of radiomics, genomics, and AI in meningioma research encounters significant translational bottlenecks. Multimodal data fusion combined with transfer learning can enhance model generalizability, whereas spatial genomics and epigenetic investigations may provide deeper biological insights into molecular-imaging correlations. Three critical dimensions warrant future investigation: (1) establishing standardized multicenter imaging protocols to address data heterogeneity; (2) developing explainable AI models to uncover molecular drivers underlying imaging features; and (3) integrating liquid biopsy with dynamic imaging surveillance to bridge static correlations with dynamic progression patterns. Breakthroughs in this domain could advance the precision classification of meningiomas and provide valuable references for further research on other CNS tumors.

#### 4.3.3 Evolving Clinical Demands and Novel Paradigms in Meningioma MRI

MRI research in meningiomas is transitioning from diagnostic tools to intelligent decision-making systems. In preoperative diagnosis, AI-powered multiparametric models are revolutionizing traditional radiologist-dependent interpretation. The nomogram model developed by Zhao *et al*. [39], which incorporates multiparametric MRI and clinical indicators, achieved enhanced predictive performance for preoperative WHO grading (AUC = 0.885/0.860). Gui *et al*. [51] demonstrated that a radiomics-deep learning hybrid model demonstrated effective sinus invasion prediction in 601 cases (AUC 0.818/0.769). These studies collectively validate the clinical utility of multimodal features integrated with machine learning algorithms. However, they were trained exclusively on ≥3 T MRI [41], underscoring the need for ≤1.5 T-validated models in low-resource settings. Despite single-center retrospective limitations, they lay the groundwork for standardized diagnostic-therapeutic decision systems.

AI-based image navigation has demonstrated successful applications in other intracranial tumors and can potentially be adapted to meningioma surgery [52]. While conventional 3D U-Net achieves high segmentation accuracy, it requires prolonged processing time (approximately 30 minutes) [53]. The nnU-Net adaptive architecture, particularly its 2D coarse segmentation with 3D refinement approach, reduces processing time to 5 minutes while maintaining a Dice coefficient ≥84% for brain tumor image segmentation [54, 55]. However, real-time navigation still faces challenges, including latency and insufficient dynamic adaptability [56]. Computational optimization and adaptive learning strategies are being employed to address these limitations. For instance, the ARM framework incorporates a game theory-inspired arena mechanism that implements dynamic branch optimization within U-Net convolutional layers, achieving significant improvements (*p*<0.01) in multimodal medical image segmentation [57]. Furthermore, the Reinforcement Learning (RL)-based adaptive therapy framework proposed by Gallagher *et al*. [58] optimized drug scheduling in prostate cancer by dynamically adjusting tumor burden thresholds, resulting in a 50% reduction in cumulative dosage. This principle could be adapted to meningioma surgery navigation: RL-based real-time learning from intraoperative MRI feedback may enhance dynamic boundary detection accuracy (*e.g*., drift error correction) while reducing over-reliance on static preoperative planning. Nevertheless, clinical implementation encounters multifaceted challenges, particularly high-end equipment dependency, ethical concerns, and performance variability [59]. Future research priorities include: (1) developing lightweight hybrid architectures (*e.g*., ARM framework with knowledge distillation) to balance computational efficiency and accuracy; (2) constructing multimodal intraoperative feedback systems for dynamic boundary drift correction *via* incremental learning; and (3) establishing standardized human-AI collaboration protocols with defined decision boundaries to address ethical concerns. Implementing these technological pathways may transform meningioma surgical navigation from static preoperative imaging-based planning to intelligent paradigms featuring real-time feedback and adaptive decision-making capabilities.

MRI radiomics demonstrates substantial value in postoperative assessment of meningiomas. Integration with WHO CNS5 (2021) criteria and molecular markers (*e.g*., CDKN2A/B deletion [60] and TERT promoter mutations [61]) improves prognostic stratification for high-grade meningiomas [62]. The multiparametric MRI model developed by Song *et al*. [63] (C-index = 0.845) optimized adjuvant therapeutic decision-making. This integrated predictive model based on multiparametric MRI radiomics provides enhanced prognostic capability. Furthermore, MRI radiomics increases sensitivity for residual tumor detection and enables prediction of postoperative progressive cerebral edema and hemorrhage (PPCEH) [64]. These technologies not only reveal occult heterogeneity in the tumor microenvironment but also establish quantitative criteria for personalized follow-up protocols, gradually replacing subjective Simpson grading [65].

MRI-based personalized therapeutic systems enhance meningioma treatment strategies through multimodal data integration. For example, the deep learning model developed by Chen *et al*. [66] deciphers molecular features from T1-weighted contrast-enhanced images to noninvasively predict WHO classification (AUC = 0.966) and Ki-67/H3K27me3/PR biomarker expression, thereby identifying optimal candidates for targeted therapies [67]. PR-positive tumors demonstrate enhanced sensitivity to hormone-targeted therapies. Progesterone antagonists markedly suppress WNT pathway activity in PR-positive meningiomas [68]. The deep transfer learning-based SVM model pioneered by Gao *et al*. [69] achieves the first noninvasive prediction of PR expression from MRI, providing a robust tool for preoperative evaluation of hormonal receptor status. This finding strongly aligns with clinical evidence reported by Reuter *et al*. [70], demonstrating that PR-positive tumors may undergo regression through hormonal intervention. The integration of these approaches provides critical insights for developing precision “imaging-molecular-therapy” strategies in meningioma management.

AI research for meningioma MRI demonstrates a significant imbalance between technological advancement and clinical translation. Despite demonstrating excellent diagnostic performance, AI models exhibit marked clinical implemen-tation delays. This implementation gap manifests primarily in three aspects: first, the protracted certification process for clinical deployment starkly contrasts with the rapid pace of research iterations [71]. Second, Petersen *et al*. [72] demonstrated that performance gaps of AI models across demographic groups largely stem from training data that lack representativeness and diversity, underscoring the ‘data-diversity deficit’ in medical imaging AI. Third, pediatric meningioma AI research remains scarce despite its distinct molecular/MRI profiles from adults, highlighting the need for age-specific studies and prospective research designs. The global disparity in high-end MRI availability exacerbates AI research inequalities. Studies reveal staggering disparities [37, 73]: high-income countries possess 26.53 MRI units/million population (≥3T), versus merely 1.12 units/million (<1.5T) in LMICs. This discrepancy creates an AI dilemma: while current algorithms predominantly utilize 3T data from high-income countries, LMICs primarily employ low-field MRI systems with substantially different Signal-To-Noise Ratios (SNR). Such a “technology-data mismatch” may widen global diagno-stic disparities. Additionally, stringent privacy regulations coupled with inadequate data-sharing frameworks constrain multicenter collaborative research. Addressing these systemic challenges requires establishing interdisciplinary collaborative innovation systems.

Bridging these disparities necessitates a paradigm shift toward “equitable AI.” Key strategies encompass: Federated learning with differential privacy simultaneously ensures regulatory compliance and multicenter data integration. Hardware-adaptive algorithms (*e.g*., unsupervised domain adaptation for MRI field-strength conversion) improve cross-platform robustness. Achieving equity in meningioma MRI AI requires addressing data diversity and technological access simultaneously. While global consortia must establish diversity standards for training data, parallel efforts to promote accessible technologies like low-field MRI and local AI training paradigms (*e.g*., federated learning) are essential to ensure these tools are viable and effective in low-resource settings [74]. These initiatives require coordinated multilateral efforts. Only through such systematic integration can AI's transformative potential in meningioma MRI align with the ethical imperatives of universal healthcare.

To ground the “equitable AI” claim in verifiable data, we note that the 2021 systematic review by Ugga *et al*. [7], covering 25 AI/radiomics studies, did not identify any model explicitly validated on ≤1.5 T scanners. The development of meningioma MRI AI is constrained by a global “technology-data imbalance,” where the predominance of high-field scanners and curated datasets in high-income countries creates a closed-loop validation ecosystem with poor generalizability to low-resource settings that rely on older or lower-field equipment [75]. This study is the first to quantify this equity gap using bibliometrics and to propose a field-strength-agnostic, three-dimensional framework for equitable AI deployment, thereby aligning AI's transformative potential with the ethical imperative of universal health coverage.

## 5 LIMITATIONS

Although this systematic bibliometric analysis elucidates evolutionary patterns in meningioma MRI research, several limitations warrant consideration. First, exclusive reliance on WoSCC may omit significant contributions from non-English publications and regional databases, potentially biasing the assessment of the global research landscape. Second, algorithmic heterogeneity across analytical tools may introduce subtle variations in the interpretation of keyword networks.

## CONCLUSION

This bibliometric study delineates the evolutionary arc of meningioma MRI from morphological imaging to AI-driven precision. Three paradigm shifts, structural, functional, and intelligent, have improved diagnostic accuracy, yet multicenter standardization, AI interpretability, and resource equity remain limited. Future priorities include harmonized protocols, explainable AI, and lightweight algorithms compatible with low-field scanners. By integrating burst-sigma mapping with global equipment metrics, we provide quantitative evidence that the temporal and field-strength gaps between high-impact guidelines and AI algorithm deployment exceed those previously described, and outline a data-driven, field-strength-agnostic framework for neuro-oncological imaging.

## Figures and Tables

**Fig. (2):**
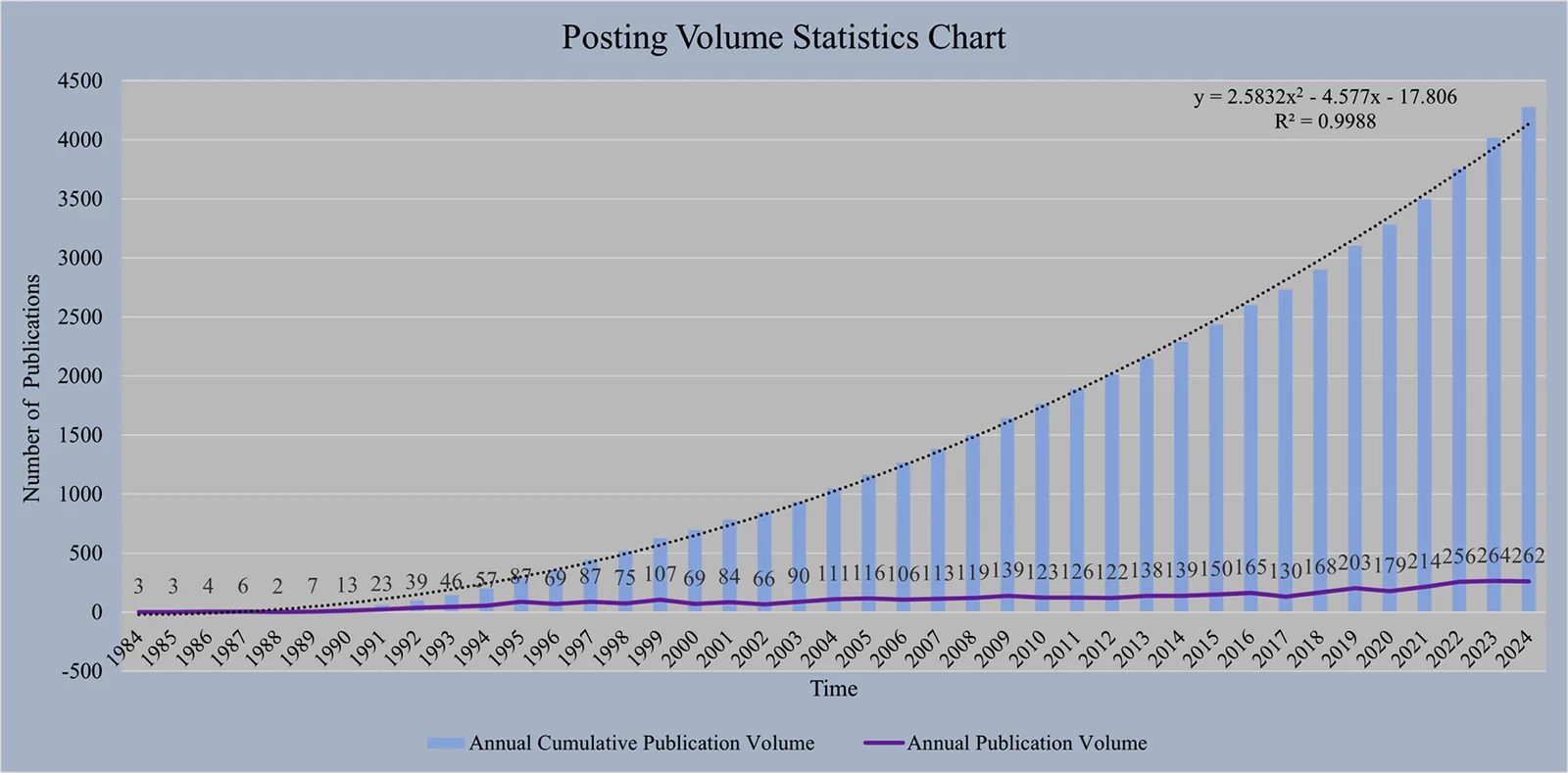
**Posting volume statistics chart.** The black dashed quadratic curve (y = 2.5832x^2^ – 4.577x – 17.806, R^2^ = 0.9988) indicates an S-shaped technology-diffusion pattern. Three phases are marked: (i) 1984–2000 slow uptake (CAGR ≈ 2.8%), (ii) 2001–2019 rapid expansion (CAGR ≈ 14.6%), (iii) 2020–2024 maturity yet record output (61.7% of the 40-year corpus published within these five years), coinciding with the AI-driven imaging boom.

**Fig. (4a):**
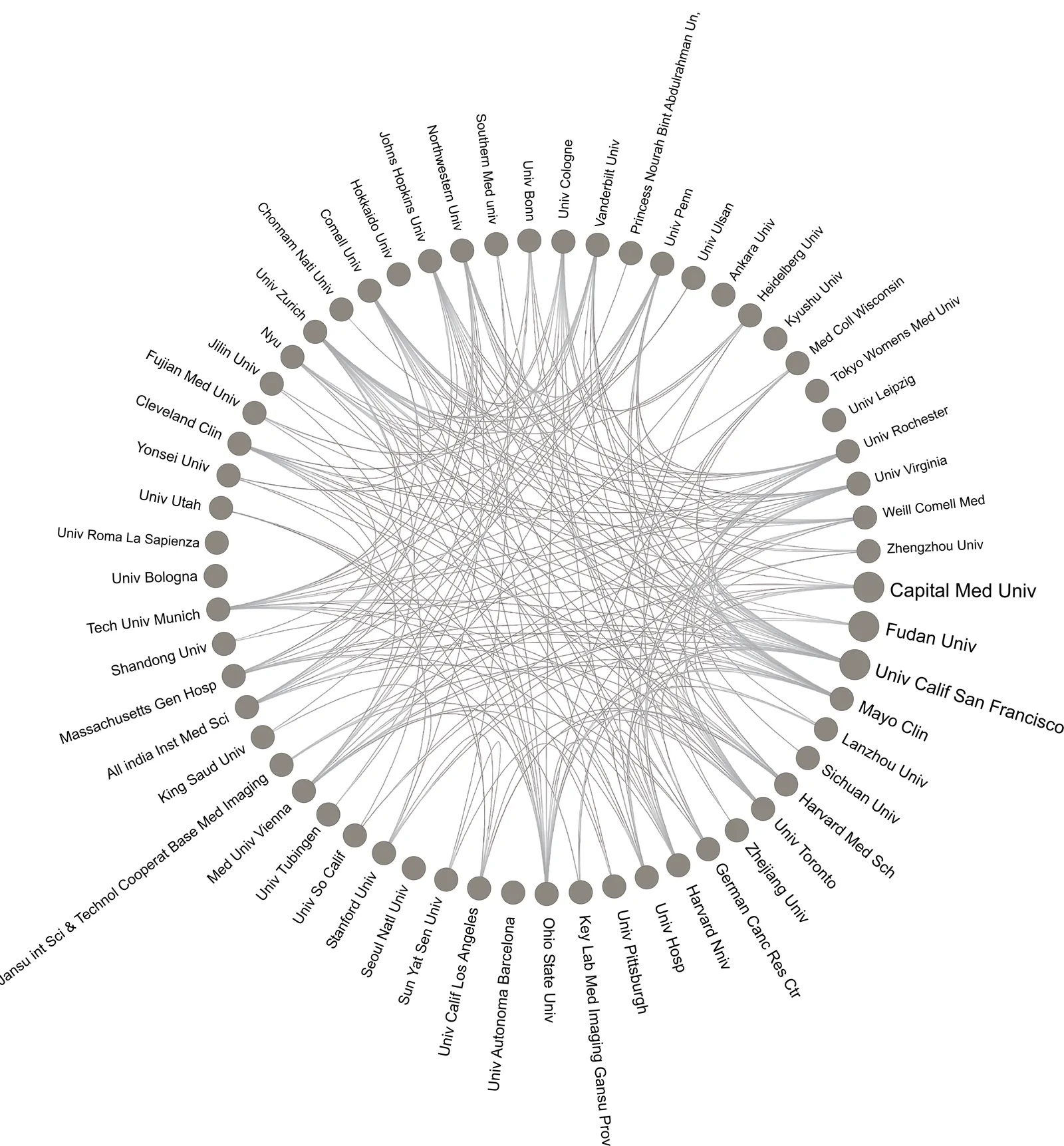
**Diagram of the institutional research cooperation network.** Institutional network: node size = fractional paper output; edges represent co-authorship links (threshold ≥ 3 joint publications). Harvard University forms the largest hub, bridging North American and Eurasian clusters.

**Fig. (4b):**
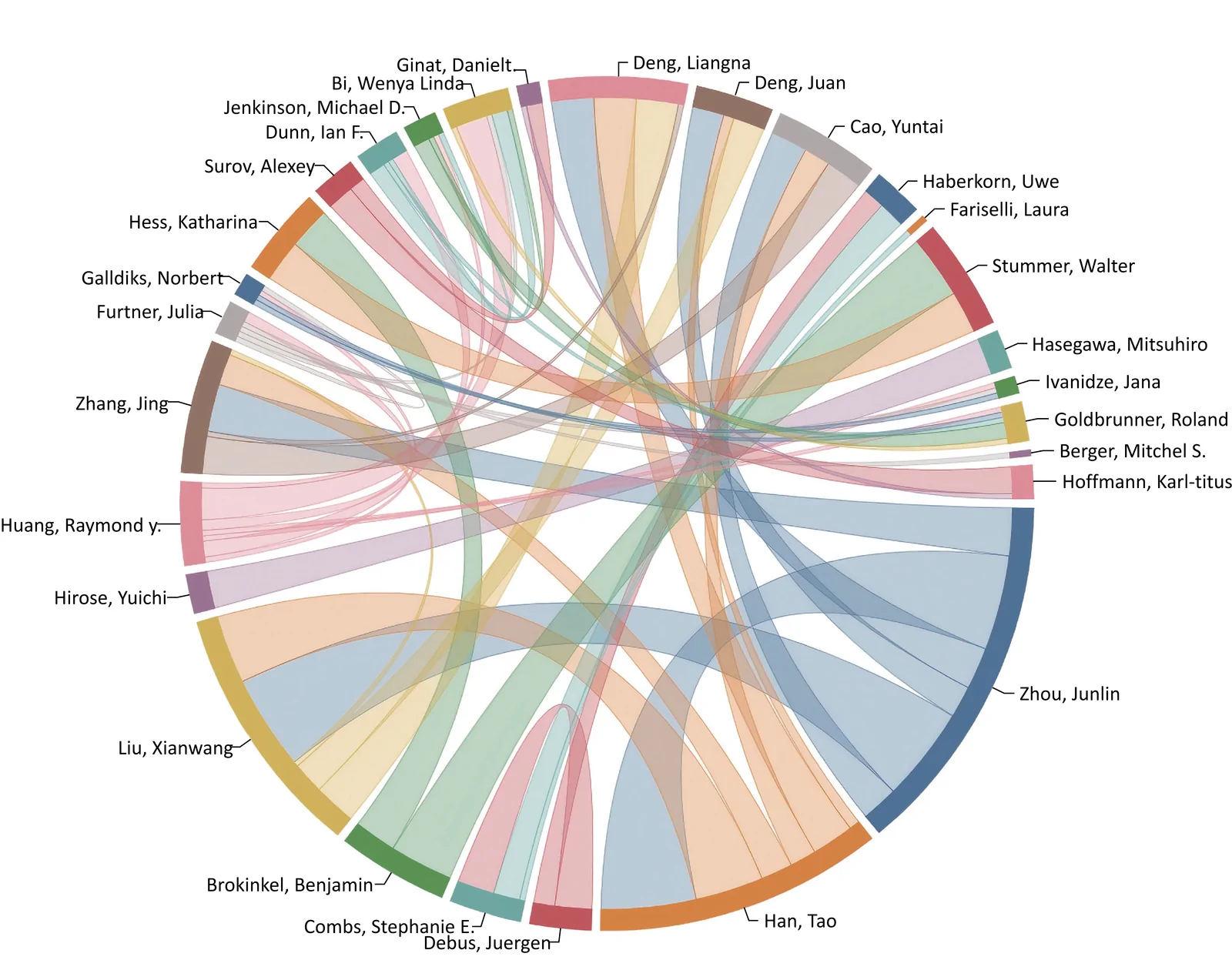
**Author collaborates on network chord diagrams.** Author chord diagram: arc length = individual publication count; ribbon thickness = number of collaborative papers. Three dominant communities (radio-oncology, neuropathology, and AI segmentation) are color-coded and interconnected by high-betweenness brokers.

**Fig. (6a):**
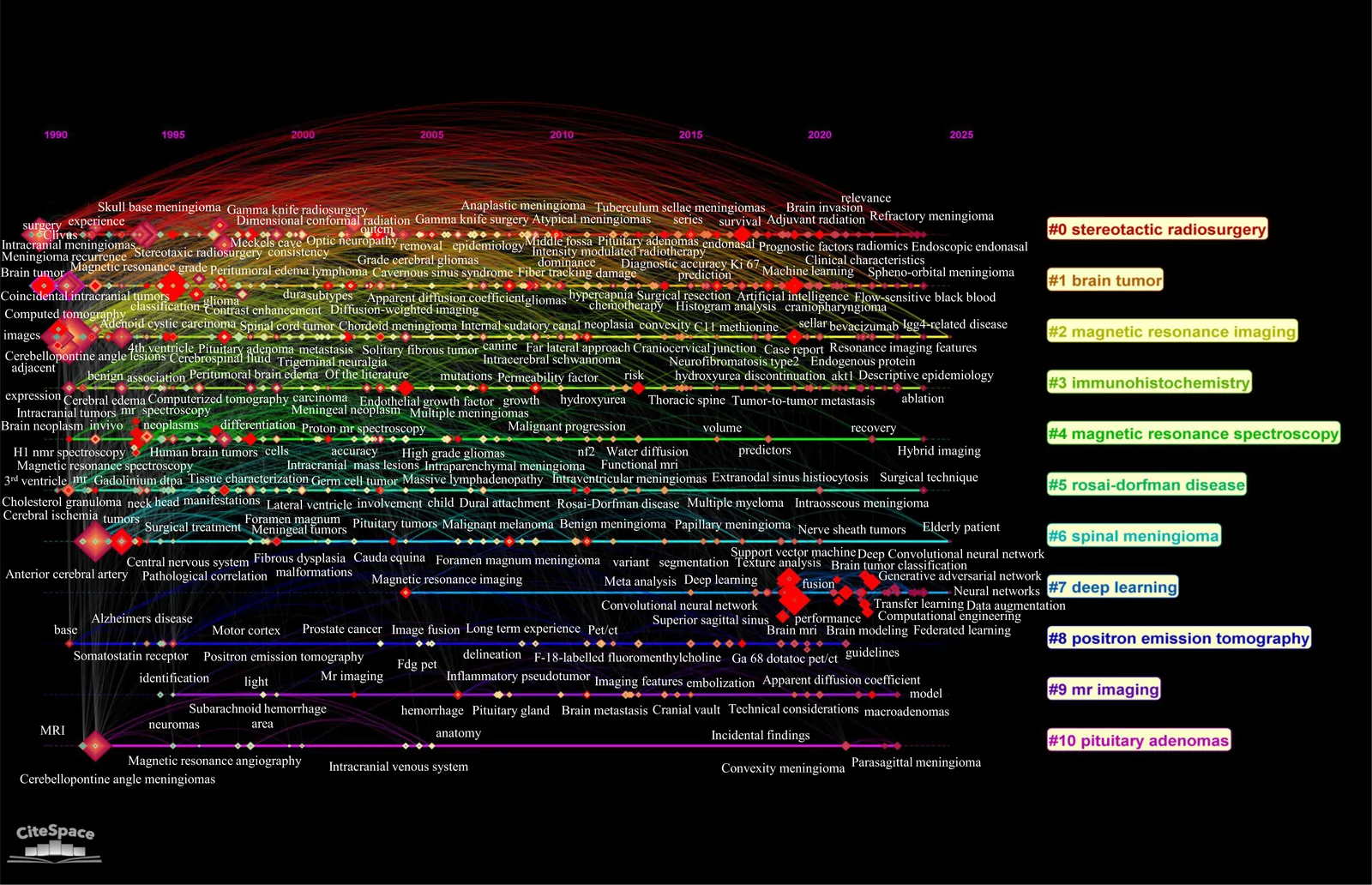
**CiteSpace timeline view of keyword co-occurrence (top 50 per 2-year slice, 1984–2024).** Node size = frequency; color band = first-appearance year. Early clusters (1990s) center on “MRI” and “meningioma”, whereas post-2018 layers are dominated by “deep learning”, “radiomics”, and “segmentation”, visualizing the field’s shift from structural imaging to AI-driven analysis.

**Fig. (6b):**
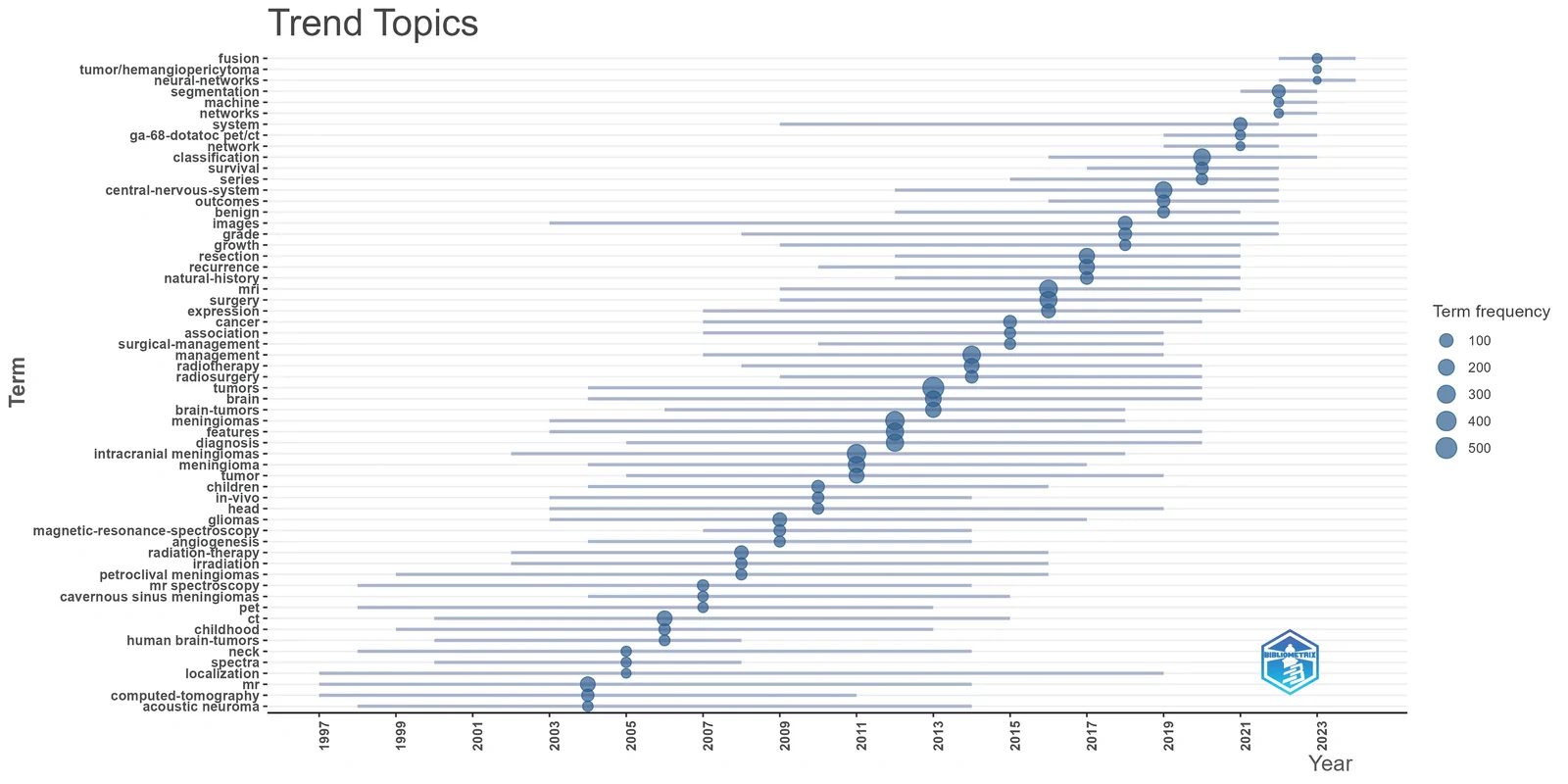
**Temporal evolution of medical terms in meningioma-MRI literature (1997–2023).** Each row represents one term; the dot diameter is proportional to its within-year frequency. “Ki-67”, “DWI”, and “CNN” show monotonic upward trajectories, reflecting the sequential adoption of molecular, functional, and AI-based research foci.

**Fig. (6c):**
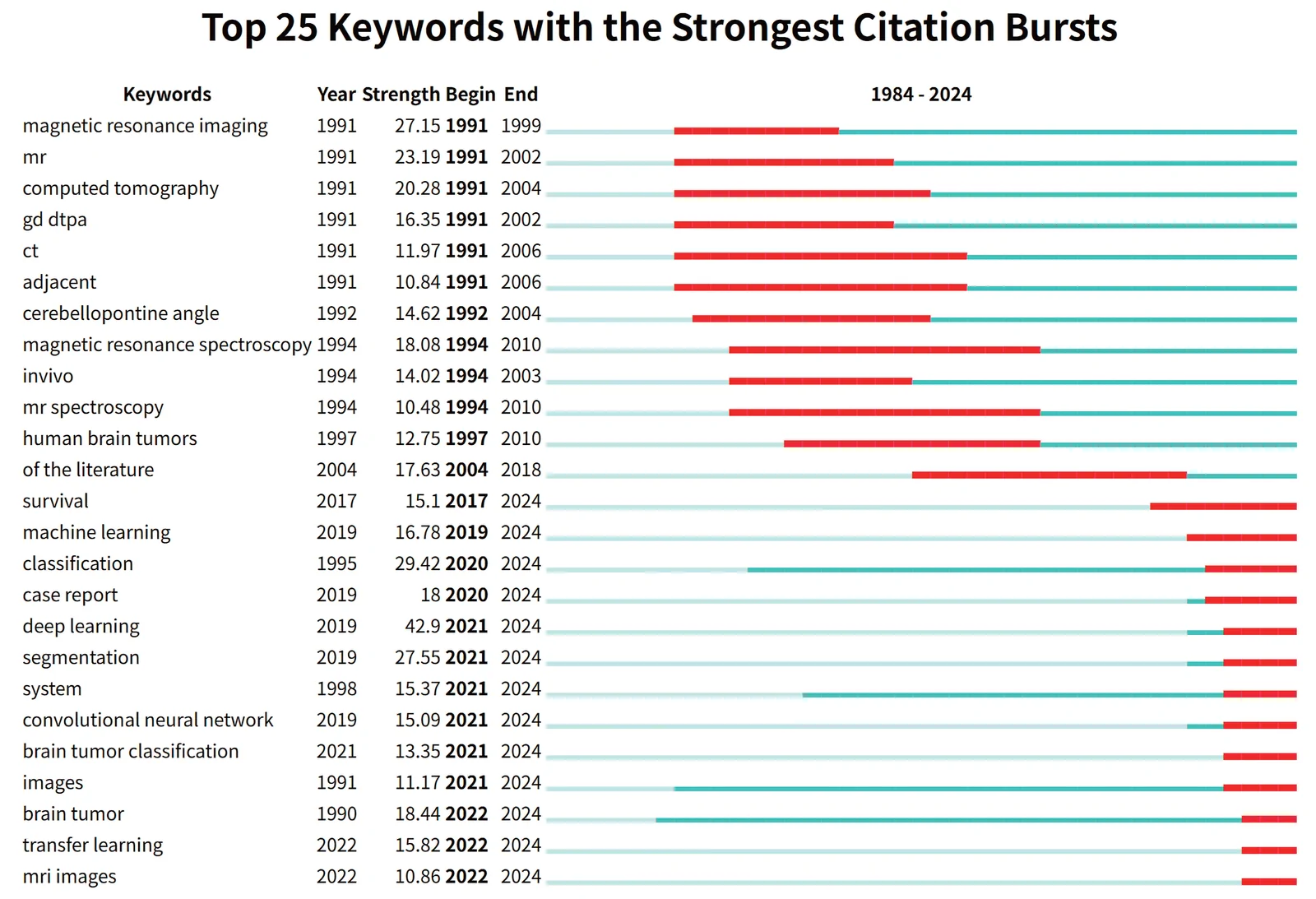
**Top-25 citation-burst keywords (1984–2024) computed by CiteSpace (γ = 0.3, minimum burst duration = 3 years).** “Deep learning” exhibits the strongest burst (strength = 42.9, 2018–2022), followed by “segmentation” (38.7, 2017–2021), signaling the recent AI-driven paradigm shift. Bar length denotes burst duration; onset year is labelled at the leading edge.

**Fig. (6d):**
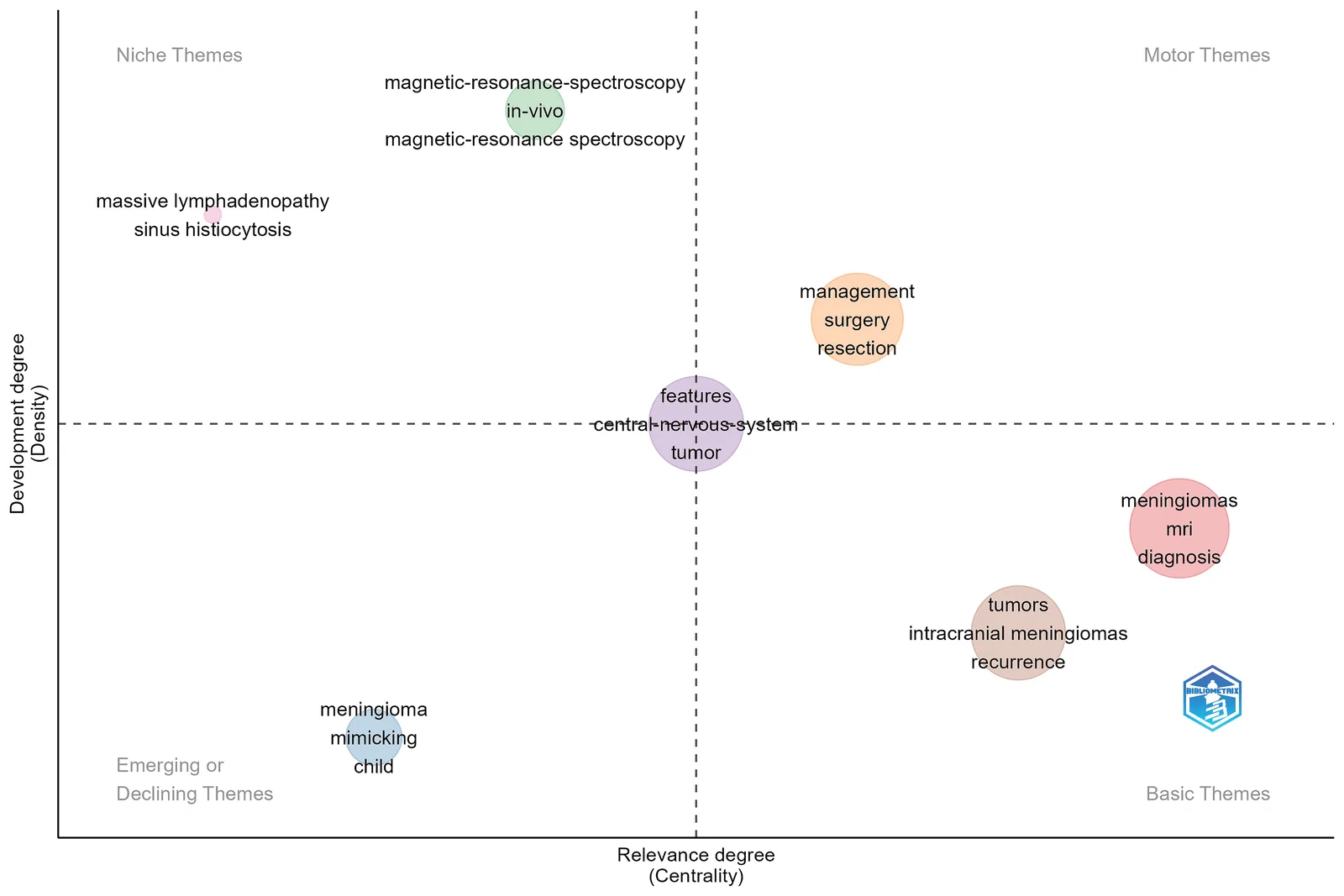
**Strategic diagram plotting thematic maturity versus centrality for 11,179 unique keywords (1984–2024). x-axis** = betweenness centrality (relevance); **y-axis** = clustering density (development). **Quadrant labels:** – upper-right = **core drivers** (*e.g*., “CNS”, “features”) sustaining knowledge hubs; – lower-right = **emerging or declining** topics drifting toward the core, exemplified by “meningioma-mimicking lesions” and “pediatric meningioma, indicating imminent research frontiers.

**Fig. (7):**
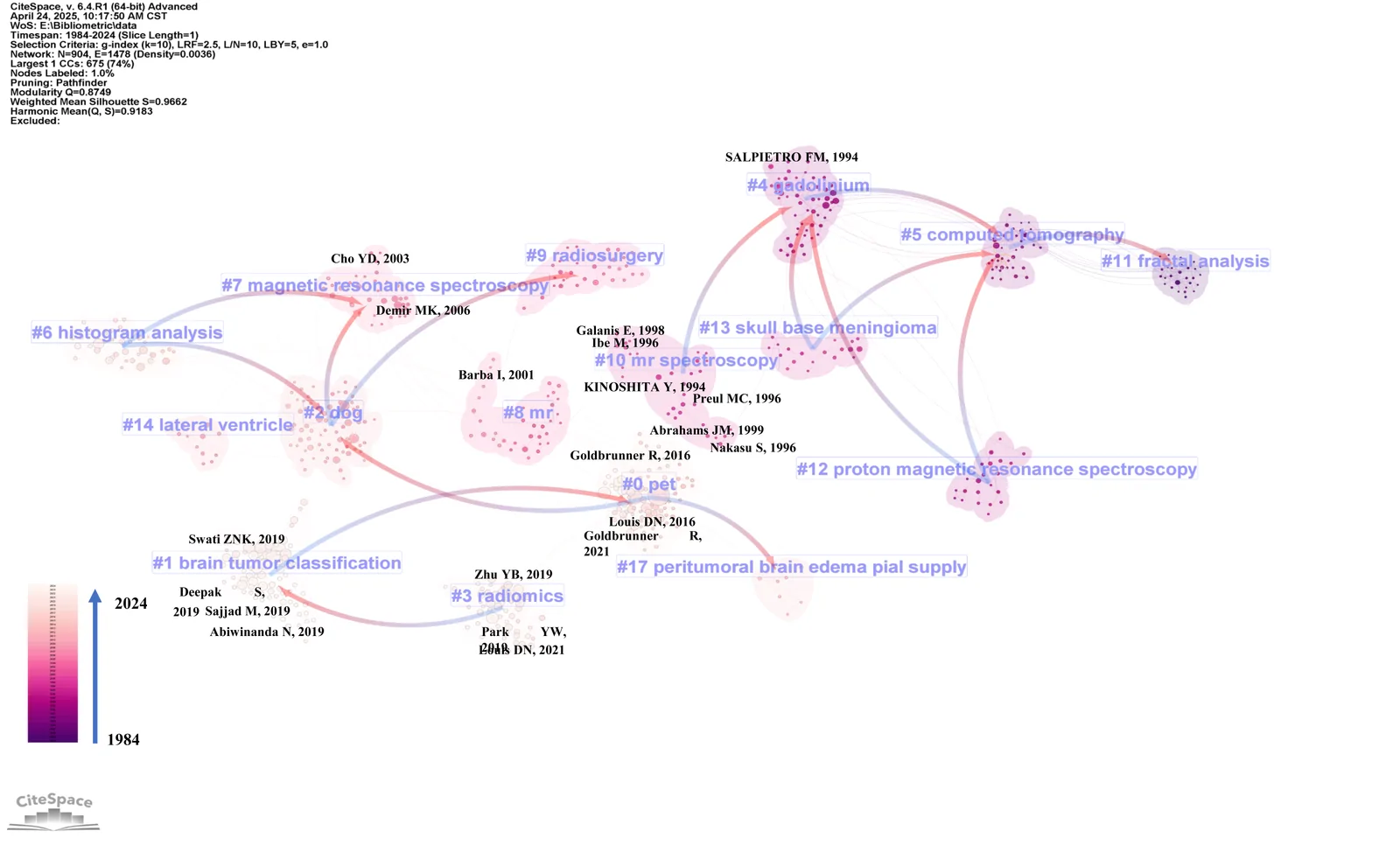
**Co-citation network (16 clusters, 1984–2024) visualizing the intellectual backbone of meningioma-MRI research.** Node diameter ∝ co-citation frequency; edge thickness α co-citation strength; colour gradient = publication year. **Cluster 0** (Louis DN 2016) forms the root, bridging molecular taxonomy to imaging. High-betweenness nodes (Goldbrunner 2016, Deepak 2019) link radio-oncology guidelines to AI segmentation methods, revealing the field’s evolution from histopathological grading to deep-learning-assisted diagnosis.

**Table 1:** Search formula compilation table.

Search Query Number	Search Query
#1	TS=(“Meningioma” OR “Meningiomas” OR “Meningiomatosis” OR “Benign Meningioma” OR “Malignant Meningioma” OR “Atypical Meningioma” OR “Anaplastic Meningioma” OR “Grade I Meningioma” OR “Grade II Meningioma” OR “Grade III Meningioma” OR “Clear Cell Meningioma” OR “Fibrous Meningioma” OR “Papillary Meningioma” OR “Psammomatous Meningioma” OR “Transitional Meningioma” OR “Secretory Meningioma” OR “Intracranial Meningioma” OR “Skull Base Meningioma” OR “Spinal Meningioma” OR “Intraventricular Meningioma” OR “Parasagittal Meningioma” OR “Sphenoid Wing Meningioma” OR “Olfactory Groove Meningioma”)
#2	TS= (“Magnetic Resonance Imaging” OR MRI OR “Functional MRI” OR fMRI OR “Chemical Shift Imaging” OR “Spin Echo Imaging” OR “Magnetic Resonance Spectroscopy” OR MRS OR “Magnetization Transfer Imaging” OR “Diffusion Tensor Imaging” OR DTI OR “Diffusion MRI” OR “Magnetic Resonance Angiography” OR MRA OR “MRI Scan” OR “DCE” OR “Dynamic Contrast Enhanced” OR “DSC” OR “Dynamic Susceptibility Contrast” OR “ASL” OR “Arterial Spin Labeling” OR “SWI” OR “Susceptibility Weighted Imaging” OR “FLAIR” OR “Fluid Attenuated Inversion Recovery” OR “Apparent Diffusion Coefficient” OR ADC)
#1 AND #2	TS=((“Meningioma” OR “Meningiomas” OR “Meningiomatosis” OR “Benign Meningioma” OR “Malignant Meningioma” OR “Atypical Meningioma” OR “Anaplastic Meningioma” OR “Grade I Meningioma” OR “Grade II Meningioma” OR “Grade III Meningioma” OR “Clear Cell Meningioma” OR “Fibrous Meningioma” OR “Papillary Meningioma” OR “Psammomatous Meningioma” OR “Transitional Meningioma” OR “Secretory Meningioma” OR “Intracranial Meningioma” OR “Skull Base Meningioma” OR “Spinal Meningioma” OR “Intraventricular Meningioma” OR “Parasagittal Meningioma” OR “Sphenoid Wing Meningioma” OR “Olfactory Groove Meningioma”) AND (“Magnetic Resonance Imaging” OR MRI OR “Functional MRI” OR fMRI OR “Chemical Shift Imaging” OR “Spin Echo Imaging” OR “Magnetic Resonance Spectroscopy” OR MRS OR “Magnetization Transfer Imaging” OR “Diffusion Tensor Imaging” OR DTI OR “Diffusion MRI” OR “Magnetic Resonance Angiography” OR MRA OR “MRI Scan” OR “DCE” OR “Dynamic Contrast Enhanced” OR “DSC” OR “Dynamic Susceptibility Contrast” OR “ASL” OR “Arterial Spin Labeling” OR “SWI” OR “Susceptibility Weighted Imaging” OR “FLAIR” OR “Fluid Attenuated Inversion Recovery” OR “Apparent Diffusion Coefficient” OR ADC))

**Table 2:** Table of top 25 countries by number of publications.

**Country**	**H_index**	**G_index**	NP*	TC*	PY_start*	CPP/year*	MNCS*
USA	117	186	2465	77383	1984	1.20	1.05
CHINA	65	84	1828	21933	1992	0.58	0.51
GERMANY	100	152	1248	42283	1987	1.01	0.89
JAPAN	64	106	1087	21669	1984	0.58	0.51
ITALY	52	93	673	15541	1985	0.74	0.65
FRANCE	63	91	672	15278	1990	0.75	0.66
UNITED KINGDOM	66	119	557	20248	1985	1.01	0.89
INDIA	36	66	496	6630	1993	0.58	0.51
KOREA	40	64	437	7499	1998	0.78	0.69
TURKEY	35	54	340	4882	1993	0.69	0.61
SPAIN	63	102	326	12912	1992	1.15	1.01
CANADA	41	75	220	6777	1991	1.01	0.89
SWITZERLAND	37	75	206	6569	1991	1.40	1.23
NETHERLANDS	34	94	180	9469	1993	1.83	1.61
SAUDI ARABIA	28	43	168	2413	1989	0.54	0.47
AUSTRIA	29	58	146	3804	1991	1.01	0.89
SWEDEN	42	80	139	6856	1994	1.31	1.15
AUSTRALIA	26	45	136	2630	1995	0.78	0.69
BELGIUM	34	57	126	3571	1991	1.01	0.89
IRAN	25	50	120	2857	2000	0.91	0.80
POLAND	16	30	117	1184	1995	0.69	0.61
GREECE	23	49	103	2643	1996	0.91	0.80
PAKISTAN	28	48	99	2499	2016	2.84	2.49
BRAZIL	14	26	97	1050	1998	0.78	0.69
DENMARK	26	45	81	2424	1991	1.15	1.01

**Note:** NP*:Total number of publications TC*; Total citation frequency PY_start*; The year of the publication; CPP/year*: TC* ÷ NP* ÷ (2024 − PY_start* +1); MNCS: The normalization is performed using the global mean of the “Neuroimaging & Neurosurgery” subcategory released in WoS 2024-06 (i.e., MNCS = CPP ÷ the global average CPP of this subcategory, with a 2024-06 baseline of 11.4).

**Table 3:** Top 20 institutions by publication volume - statistical table.

**Institution**	**H_index**	**G_index**	NP*	TC*	PY_start*	CPP/year*	MNCS*
UNIVERSITY OF CALIFORNIA SYSTEM	53	85	210	8324	1989	1.20	1.05
HARVARD UNIVERSITY	56	111	178	12932	1991	1.83	1.61
FUDAN UNIVERSITY	23	34	123	1650	2006	0.91	0.80
UNIVERSITY OF LONDON	40	66	119	4758	1989	1.20	1.05
MAYO CLINIC	24	47	117	2578	1993	0.91	0.80
CORNELL UNIVERSITY	27	50	115	2921	1985	1.01	0.89
ASSISTANCE PUBLIQUE HOPITAUX PARIS (APHP)	33	60	113	3934	1992	1.15	1.01
UNIVERSITE PARIS CITE	29	55	103	3371	1992	1.15	1.01
CAPITAL MEDICAL UNIVERSITY	14	24	94	836	2003	0.58	0.51
HELMHOLTZ ASSOCIATION	37	64	93	4315	1993	1.31	1.15
WEILL CORNELL MEDICINE	23	47	93	2432	2008	1.15	1.01
HARVARD MEDICAL SCHOOL	39	88	88	8286	1991	1.83	1.61
UNIVERSITY OF CALIFORNIA SAN FRANCISCO	35	64	86	4229	1989	1.20	1.05
RUPRECHT KARLS UNIVERSITY HEIDELBERG	34	66	81	4374	1992	1.31	1.15
UNIVERSITY OF TORONTO	28	63	81	4025	1993	1.31	1.15
EBERHARD KARLS UNIVERSITY OF TUBINGEN	24	54	78	2949	1987	1.20	1.05
UNIVERSITY SYSTEM OF OHIO	18	34	72	1333	1995	0.78	0.69
SEOUL NATIONAL UNIVERSITY (SNU)	16	25	70	830	1999	0.78	0.69
GERMAN CANCER RESEARCH CENTER (DKFZ)	34	60	68	3633	1993	1.31	1.15
SICHUAN UNIVERSITY	15	27	68	913	2008	0.91	0.80

**Note:** NP*:Total number of publications TC*; Total citation frequency PY_start*; The year of the publication; CPP/year*: TC* ÷ NP* ÷ (2024 − PY_start* +1); MNCS: The normalization is performed using the global mean of the “Neuroimaging & Neurosurgery” subcategory released in WoS 2024-06 (i.e., MNCS = CPP ÷ the global average CPP of this subcategory, with a 2024-06 baseline of 11.4).

**Table 4:** Statistics table of the top 30 authors by publication volume.

Authorship	**H_index**	**G_index**	**M_index**	NP*	TC*	PY_start*
ZHOU JL	9	15	0.643	31	271	2012
ZHANG J	10	18	0.333	30	347	1996
ZHANG Y	10	17	0.667	24	304	2011
WANG Y	10	13	0.5	23	205	2006
DEBUS J	17	22	0.515	22	1574	1993
KIM JH	12	20	0.522	21	404	2003
HAN T	6	11	1	20	140	2020
LIU XW	5	7	0.625	19	70	2018
ARÚS C	14	17	0.5	17	1372	1998
LI Y	8	14	0.5	17	197	2010
SAMII M	13	16	0.433	16	938	1996
NAKAMURA M	10	16	0.345	16	530	1997
TONN JC	10	16	0.4	16	686	2001
WANG L	9	16	0.529	16	367	2009
ZHANG H	8	16	0.444	16	289	2008
SCHWARTZ TH	11	15	0.688	15	468	2010
SUROV A	14	14	1.273	14	839	2015
KURISU K	10	14	0.313	14	736	1994
KIM SH	9	14	0.333	14	375	1999
GENG DY	8	14	0.444	14	247	2008
JUNG S	7	14	0.333	14	196	2005
KIM S	6	14	0.4	14	324	2011
COMBS SE	12	13	0.8	13	572	2011
ARITA K	9	13	0.281	13	688	1994
STUMMER W	9	13	1.125	13	198	2018
MAJÓS C	12	12	0.444	12	1177	1999
BROKINKEL B	8	12	1	12	185	2018
MCDERMOTT MW	8	12	0.364	12	293	2004
YAMAGUCHI S	8	12	0.348	12	153	2003
LEE SK	7	12	0.206	12	284	1992

**Note:** NP*:Total number of publications TC*:Total citation frequency PY_start*: The year of the publication.

**Table 5:** Statistical table of the top 17 journals published in the core area.

**Source**	NP*	TC*	PY_start*
WORLD NEUROSURGERY	217	2043	2010
JOURNAL OF NEUROSURGERY	129	5015	1987
NEUROSURGERY	128	6699	1990
NEURORADIOLOGY	117	3007	1987
ACTA NEUROCHIRURGICA	117	2916	1986
JOURNAL OF NEURO-ONCOLOGY	98	2403	1993
JOURNAL OF CLINICAL NEUROSCIENCE	81	1106	1997
CLINICAL NEUROLOGY AND NEUROSURGERY	77	882	1994
NEUROLOGIA MEDICO-CHIRURGICA	69	732	1998
SURGICAL NEUROLOGY	65	2176	1986
NEUROLOGICAL SURGERY	55	295	1995
FRONTIERS IN ONCOLOGY	53	417	2019
NEUROCHIRURGIE	52	457	1994
NEUROSURGICAL REVIEW	50	1013	1994
BRITISH JOURNAL OF NEUROSURGERY	50	772	1993
CHILDS NERVOUS SYSTEM	49	560	1995
EUROPEAN RADIOLOGY	48	1421	1995

**Note:** NP*:Total number of publications TC*:Total citation frequency PY_start*: The year of the publication.

**Table 6:** Key Co-cited references in meningioma MRI research.

**Rank**	**Reference (Author, Year, Journal)**	**Citations**	**Research Focus**	**Methodological Contribution**
1	Louis DN, 2016, Acta Neuropathol	12208	Histopathology-MRI characteristics in WHO's first molecular integration classification	Establish a grading standard for linking tumor-brain interface enhancement characteristics with molecular indicators such as IDH and H3 K27M (Evidence Level: Level III)
2	Louis DN, 2021, Neuro-Oncology	6694	Molecular biomarkers and MRI correlations	Introducing a multimodal fusion model of DNA methylation sequencing and imagingomics
3	Goldbrunner R, 2016, Lancet Oncol	628	MRI-based clinical decision-making pathway optimization	Guidelines for establishing treatment strategy stratification
4	Deepak S, 2019, Comput Biol Med	590	The universal solution of cross-domain transfer learning in the context of MRI data scarcity	End-to-end fine-tuning system based on GoogLeNet
5	Sajjad M, 2019, J Comput Sci-Neth	442	Adversarial data augmentation techniques for the lack of MRI data	CycleGAN is introduced to generate multimodal synthesis data
6	Swati ZNK, 2019, Comput Med Imag Grap		The block fine-tuning strategy optimizes the performance of CNNs in small sample data	Developing a modular migration framework: ImageNet pre-training-VGG16 layer block adaptive learning
7	Goldbrunner R, 2021, Neuro-Oncology	391	Controversy over the criteria for quantitative evaluation of postoperative residual volume	Define the critical value of postoperative residuals for “intervention” (volume ≥5cm^3^ or adjacent to critical functional areas)
8	Rehman A, 2020, Circuits, Systems, and Signal Processing	306	A comparative study of deep transfer learning architecture	Hyperparameter optimization strategy for VGG16 fine-tuning model
9	Sultan, HH, 2019, IEEE ACCESS	298	Multicenter MRI Data Compatibility Solution	A cross-dataset feature normalization protocol is proposed
10	Abiwinanda N, 2019, IFMBE Proc	292	Developing Simplified CNN Classification Brain Tumor MRI	Verification accuracy of single convolutional layer structure without presegment reached 84.19%
11	Badza MM, 2020, Applied Sciences-Basel	257	Lightweight CNNs enable accurate classification of non-invasive brain tumors (three types).	The newly designed lightweight CNN has both high generalization capability and real-time performance through enhanced data and cross-validation
12	Park YW, 2019, Eur Radiol	152	Multimodal MRI imaging synthesis achieves non-invasive grading distinction before surgery for meningioma	Multiparameter machine learning model fusion of full tumor volume characteristics to improve meningioma grading and subtype prediction
13	Zhu YB, 2019, Eur J Radiol	121	Conventional MRI+DLR noninvasive meningioma grading	DLR model (AUC 0.811) transcends traditional methods to improve the grading accuracy of non-invasive meningiomas

## LIST OF ABBREVIATIONS

ADC: Apparent Diffusion Coefficient
AI: Artificial Intelligence
APC: Article Processing Charge
APHP: Assistance Publique – Hôpitaux de Paris
CAGR: Compound Annual Growth Rate
CNN: Convolutional Neural Network
CPP: Citations per Paper
DCE: Dynamic Contrast Enhanced
DSC: Dynamic Susceptibility Contrast
DTI: Diffusion Tensor Imaging
DWI: Diffusion Weighted Imaging
EANO: European Association of Neuro-Oncology
Fmri: Functional Magnetic Resonance Imaging
FLAIR: Fluid Attenuated Inversion Recovery
LMICs: Low- and Middle-Income Countries
MNCS: Mean Normalized Citation Score
MRI: Magnetic Resonance Imaging
MRS: Magnetic Resonance Spectroscopy
MTR: Magnetization Transfer Ratio
Nn: Number needed to cite
RL: Reinforcement Learning
SWI: Susceptibility Weighted Imaging
WHO: World Health Organization
WoS: Web of Science
WoSCC: Web of Science Core Collection
