# Supplementary material for: MRI and Meningioma Research: Hotspots and Trends via Bibliometric Analysis
Source: Curr Med Imaging. 2026 Feb 2;22:e15734056425234. doi: 10.2174/0115734056425234251229184318 (PMC13523180; doi:10.2174/0115734056425234251229184318)
Supplement: Supplementary file 1 [file CMIM-22-E15734056425234_SD1.pdf]

1875-6603/26 *Send Orders for Reprints to reprints@benthamscience.net*

BENTHAM SCIENCE

Current Medical Imaging

Content list available at: https://benthamscience.com/journals/cmir

Supplementary Material

MRI and Meningioma Research: Hotspots and Trends *via* Bibliometric Analysis

FuQiang Tang[1], Zhou yuan Wei[1], Xuan Su[1], Limei Hou[3], Ming Yang[4] and Jing Zhang[2,*]

[1]*Department of Nursing, Zhuhai Campus of Zunyi Medical University, Zhuhai, China*
[2]*Department of Radiology, The Fifth Affiliated Hospital of Zunyi Medical University, Zhuhai, China*
[3]*Department of General Medicine, The Fifth Affiliated Hospital of Zunyi Medical University, Zhuhai, China*
[4]*Wuhan Blood Center, Wuhan, China*

SUPPLEMENTARY MATERIAL 1

Time of creation: Tue Apr 22 08:48:10 CST 2025

Introduction

This summary is automatically generated. To maximize the level of details in the summary, complete the following tasks before running this function:

- clustering and labeling by using the **All in One** button
- run the burst detection function from the **Burstness** tab in the Control Panel
- compute the betweenness centrality from the Nodes menu
- save the current visualization as a PNG image in the project folder

The summary highlights major clusters first, including citing articles and cited references. The importance of nodes will be summarized in terms of citation-based metrics such as citation counts and citation bursts, network-based metrics such as degree centrality and betweenness centrality. Sigma is a combination of both types, i.e., burst and betweenness centrality.

There are other features that are not included in the current summary, for example, structural variation analysis, analysis of uncertainties, concept trees, and dual-map overlays.

MAJOR CLUSTERS

The network consists of **11** clusters. The largest **11** clusters are summarized as follows.

Table 1. Summary of the largest 11 clusters.

ClusterID	Size	Silhouette	Label (LSI)	Label (LLR)	Label (MI)	Average Year

CITATION COUNTS

The top ranked item by citation counts is magnetic resonance imaging in Cluster #2, with citation counts of **627**. The second one is tumors in Cluster #6, with citation counts of **503**. The third is intracranial meningiomas in Cluster #0, with citation counts of **358**. The 4th is meningiomas in Cluster #2, with citation counts of **332**. The 5th is mri in Cluster #10, with citation counts of **292**. The 6th is central nervous system in Cluster #6, with citation counts of **286**. The 7th is management in Cluster #0, with citation counts of **275**. The 8th is features in Cluster #2, with citation counts of **268**. The 9th is diagnosis in Cluster #2, with citation counts of **264**. The 10th is surgery in Cluster #0, with citation counts of **252**.

Citation Counts	Node Name	Cluster ID
627	magnetic resonance imaging	2
503	tumors	6
358	intracranial meningiomas	0
332	meningiomas	2
292	mri	10
286	central nervous system	6

DOI: 10.2174/0115734056425234251229184318, 2026, *22*, e15734056425234

Citation Counts	Node Name	Cluster ID
275	management	0
268	features	2
264	diagnosis	2
252	surgery	0

BURSTS

The top ranked item by bursts is deep learning in Cluster #7, with bursts of **42.94**. The second one is classification in Cluster #1, with bursts of **29.53**. The third is segmentation in Cluster #7, with bursts of **27.58**. The 4th is magnetic resonance imaging in Cluster #2, with bursts of **27.02**. The 5th is mr in Cluster #5, with bursts of **23.23**. The 6th is computed tomography in Cluster #2, with bursts of **20.33**. The 7th is brain tumor in Cluster #1, with bursts of **19.10**. The 8th is magnetic resonance spectroscopy in Cluster #4, with bursts of **18.16**. The 9th is case report in Cluster #2, with bursts of **18.03**. The 10th is of the literature in Cluster #3, with bursts of **17.68**.

Bursts	Node Name	Cluster ID
42.94	deep learning	7
29.53	classification	1
27.58	segmentation	7
27.02	magnetic resonance imaging	2
23.23	mr	5
20.33	computed tomography	2
19.10	brain tumor	1
18.16	magnetic resonance spectroscopy	4
18.03	case report	2
17.68	of the literature	

DEGREE

The top ranked item by degree is brain in Cluster #1, with degree of **81**. The second one is brain tumor in Cluster #1, with degree of **80**. The third is ct in Cluster #5, with degree of **74**. The 4th is lesions in Cluster #2, with degree of **72**. The 5th is brain tumors in Cluster #1, with degree of **71**. The 6th is gliomas in Cluster #1, with degree of **66**. The 7th is experience in Cluster #0, with degree of **66**. The 8th is meningiomas in Cluster #2, with degree of **66**. The 9th is features in Cluster #2, with degree of **65**. The 10th is computed tomography in Cluster #2, with degree of **63**.

Degree	Node Name	Cluster ID
81	brain	1
80	brain tumor	1
74	ct	5
72	lesions	2
71	brain tumors	1
66	gliomas	1
66	experience	0
66	meningiomas	2
65	features	2
63	computed tomography	2

CENTRALITY

The top ranked item by centrality is brain tumor in Cluster #1, with centrality of **0.13**. The second one is brain tumors in Cluster #1, with centrality of **0.11**. The third is brain in Cluster #1, with centrality of **0.10**. The 4th is ct in Cluster #5, with centrality of **0.09**. The 5th is central nervous system in Cluster #6, with centrality of **0.09**. The 6th is lesions in Cluster #2, with centrality of **0.08**. The 7th is features in Cluster #2, with centrality of **0.08**. The 8th is gliomas in Cluster #1, with centrality of **0.07**. The 9th is experience in Cluster #0, with centrality of **0.07**. The 10th is meningiomas in Cluster #2, with centrality of **0.07**.

Centrality	Node Name	DOI	Cluster ID
0.13	brain tumor		1
0.11	brain tumors		1
0.10	brain		1
0.09	ct		5

Centrality	Node Name	DOI	Cluster ID
0.09	central nervous system		6
0.08	lesions		2
0.08	features		2
0.07	gliomas		1
0.07	experience		0
0.07	meningiomas		2

SIGMA

The top ranked item by sigma is brain tumor in Cluster #1, with sigma of **10.07**. The second one is mr in Cluster #5, with sigma of **4.73**. The third is computed tomography in Cluster #2, with sigma of **3.63**. The 4th is ct in Cluster #5, with sigma of **2.92**. The 5th is deep learning in Cluster #7, with sigma of **2.64**. The 6th is classification in Cluster #1, with sigma of **2.45**. The 7th is cerebellopontine angle in Cluster #2, with sigma of **2.33**. The 8th is magnetic resonance spectroscopy in Cluster #4, with sigma of **1.89**. The 9th is magnetic resonance imaging in Cluster #2, with sigma of **1.77**. The 10th is central nervous system in Cluster #6, with sigma of **1.56**.

Sigma	Node Name	DOI	Cluster ID
10.07	brain tumor		1
4.73	mr		5
3.63	computed tomography		2
2.92	ct		5
2.64	deep learning		7
2.45	classification		1
2.33	cerebellopontine angle		2
1.89	magnetic resonance spectroscopy		4
1.77	magnetic resonance imaging		2
1.56	central nervous system		6

SUPPLEMENTARY MATERIAL 2

Time of creation: Fri May 16 23:48:34 CST 2025

Introduction

This summary is automatically generated. To maximize the level of details in the summary, complete the following tasks before running this function:

- clustering and labeling by using the **All in One** button
- run the burst detection function from the **Burstness** tab in the Control Panel
- compute the betweenness centrality from the Nodes menu
- save the current visualization as a PNG image in the project folder

The summary highlights major clusters first, including citing articles and cited references. The importance of nodes will be summarized in terms of citation-based metrics such as citation counts and citation bursts, network-based metrics such as degree centrality and betweenness centrality. Sigma is a combination of both types, i.e., burst and betweenness centrality.

There are other features that are not included in the current summary, for example, structural variation analysis, analysis of uncertainties, concept trees, and dual-map overlays.

MAJOR CLUSTERS

The network consists of **16** clusters. The largest **16** clusters are summarized as follows.

Table 1. Summary of the largest 16 clusters.

ClusterID	Size	Silhouette	Label (LSI)	Label (LLR)	Label (MI)	Average Year

CITATION COUNTS

The top ranked item by citation counts is Louis DN, 2016, ACTA NEUROPATHOL, V131, P803 in Cluster #0, with citation counts of **144**. The second one is Louis DN, 2021, NEURO-ONCOLOGY, V23, P1231 in Cluster #3, with citation counts of **95**. The third is Goldbrunner R, 2016, LANCET ONCOL, V17, PE383 in Cluster #0, with citation counts of **66**. The 4th is Abiwinanda N, 2019, IFMBE PROC, V68, P183 in Cluster #1, with citation counts of **66**. The 5th is Deepak S, 2019, COMPUT BIOL MED, V111, P0 in Cluster #1, with citation counts of **62**. The 6th is Park YW, 2019, EUR RADIOL, V29, P4068 in Cluster #3, with citation counts of **61**. The 7th is Goldbrunner R, 2021, NEURO-ONCOLOGY, V23, P1821 in Cluster #0, with citation counts of **55**. The 8th is Swati ZNK, 2019, COMPUT MED IMAG GRAP, V75, P34 in Cluster #1, with citation counts of **48**. The 9th is Zhu YB, 2019, EUR J RADIOL, V116, P128 in Cluster #3, with citation counts of **47**. The 10th is Sajjad M, 2019, J COMPUT SCI-NETH, V30, P174 in Cluster #1, with citation counts of **46**.

Citation Counts	Node Name	DOI	Cluster ID
144	Louis DN, 2016, ACTA NEUROPATHOL, V131, P803	10.1007/s00401-016-1545-1	0
95	Louis DN, 2021, NEURO-ONCOLOGY, V23, P1231	10.1093/neuonc/noab106	3
66	Goldbrunner R, 2016, LANCET ONCOL, V17, PE383	10.1016/S1470-2045(16)30321-7	0
66	Abiwinanda N, 2019, IFMBE PROC, V68, P183	10.1007/978-981-10-9035-6_33	1
62	Deepak S, 2019, COMPUT BIOL MED, V111, P0	10.1016/j.compbiomed.2019.103345	1
61	Park YW, 2019, EUR RADIOL, V29, P4068	10.1007/s00330-018-5830-3	3
55	Goldbrunner R, 2021, NEURO-ONCOLOGY, V23, P1821	10.1093/neuonc/noab150	0
48	Swati ZNK, 2019, COMPUT MED IMAG GRAP, V75, P34	10.1016/j.compmedimag.2019.05.001	1
47	Zhu YB, 2019, EUR J RADIOL, V116, P128	10.1016/j.ejrad.2019.04.022	3
46	Sajjad M, 2019, J COMPUT SCI-NETH, V30, P174	10.1016/j.jocs.2018.12.003	1

BURSTS

The top ranked item by bursts is Louis DN, 2016, ACTA NEUROPATHOL, V131, P803 in Cluster #0, with bursts of **67.17**. The second one is Goldbrunner R, 2016, LANCET ONCOL, V17, PE383 in Cluster #0, with bursts of **31.15**. The third is Rogers L, 2015, J NEUROSURG, V122, P4 in Cluster #0, with bursts of **18.65**. The 4th is Louis DN, 2007, ACTA NEUROPATHOL, V114, P547 in Cluster #2, with bursts of **18.10**. The 5th is Perry A, 2007, WHO CLASSIFICATION OF TUMORS OF THE CENTRAL NERVOUS SYSTEM, V0, P164 in Cluster #2, with bursts of **18.03**. The 6th is Ostrom QT, 2014, NEURO-ONCOLOGY, V16, P1 in Cluster #0, with bursts of **16.54**. The 7th is Abiwinanda N, 2019, IFMBE PROC, V68, P183 in Cluster #1, with bursts of **16.25**. The 8th is Lin BJ, 2014, J NEUROSURG, V121, P1201 in Cluster #6, with bursts of **15.96**. The 9th is Swati ZNK, 2019, COMPUT MED IMAG GRAP, V75, P34 in Cluster #1, with bursts of **15.35**. The 10th is Deepak S, 2019, COMPUT BIOL MED, V111, P0 in Cluster #1, with bursts of **15.25**.

Bursts	Node Name	DOI	Cluster ID
67.17	Louis DN, 2016, ACTA NEUROPATHOL, V131, P803	10.1007/s00401-016-1545-1	0
31.15	Goldbrunner R, 2016, LANCET ONCOL, V17, PE383	10.1016/S1470-2045(16)30321-7	0
18.65	Rogers L, 2015, J NEUROSURG, V122, P4	10.3171/2014.7.JNS131644	0
18.10	Louis DN, 2007, ACTA NEUROPATHOL, V114, P547	10.1007/s00401-007-0278-6	2
18.03	Perry A, 2007, WHO CLASSIFICATION OF TUMORS OF THE CENTRAL NERVOUS SYSTEM, V0, P164		2
16.54	Ostrom QT, 2014, NEURO-ONCOLOGY, V16, P1	10.1093/neuonc/nou327	0
16.25	Abiwinanda N, 2019, IFMBE PROC, V68, P183	10.1007/978-981-10-9035-6_33	1
15.96	Lin BJ, 2014, J NEUROSURG, V121, P1201	10.3171/2014.7.JNS132359	6
15.35	Swati ZNK, 2019, COMPUT MED IMAG GRAP, V75, P34	10.1016/j.compmedimag.2019.05.001	1
15.25	Deepak S, 2019, COMPUT BIOL MED, V111, P0	10.1016/j.compbiomed.2019.103345	1

DEGREE

The top ranked item by degree is Goldbrunner R, 2016, LANCET ONCOL, V17, PE383 in Cluster #0, with degree of **20**. The second one is BYDDER GM, 1985, J COMPUT ASSIST TOMO, V9, P690 in Cluster #5, with degree of **18**. The third is BRANTZAWADZKI M, 1983, AM J ROENTGENOL, V140, P847 in Cluster #11, with degree of **18**. The 4th is BYDDER GM, 1982, AM J ROENTGENOL, V139, P215 in Cluster #11, with degree of **17**. The 5th is Louis DN, 2016, ACTA NEUROPATHOL, V131, P803 in Cluster #0, with degree of **15**. The 6th is Abiwinanda N, 2019, IFMBE PROC, V68, P183 in Cluster #1, with degree of **15**. The 7th is ELSTER AD, 1989, RADIOLOGY, V170, P857 in Cluster #5, with degree of **15**. The 8th is ARAKI T, 1984, RADIOLOGY, V150, P95 in Cluster #11, with degree of **15**. The 9th is Cho YD, 2003, MAGN RESON IMAGING, V21, P663 in Cluster #7, with degree of **14**. The 10th is Hwang WL, 2016, NEURO-ONCOLOGY, V18, P863 in Cluster #0, with degree of **14**.

Degree	Node Name	DOI	Cluster ID
20	Goldbrunner R, 2016, LANCET ONCOL, V17, PE383	10.1016/S1470-2045(16)30321-7	0
18	BYDDER GM, 1985, J COMPUT ASSIST TOMO, V9, P690	10.1097/00004728-198507010-00006	5
18	BRANTZAWADZKI M, 1983, AM J ROENTGENOL, V140, P847	10.2214/ajr.140.5.847	11
17	BYDDER GM, 1982, AM J ROENTGENOL, V139, P215	10.2214/ajr.139.2.215	11
15	Louis DN, 2016, ACTA NEUROPATHOL, V131, P803	10.1007/s00401-016-1545-1	0
15	Abiwinanda N, 2019, IFMBE PROC, V68, P183	10.1007/978-981-10-9035-6_33	1
15	ELSTER AD, 1989, RADIOLOGY, V170, P857	10.1148/radiology.170.3.2916043	5
15	ARAKI T, 1984, RADIOLOGY, V150, P95	10.1148/radiology.150.1.6689793	11
14	Cho YD, 2003, MAGN RESON IMAGING, V21, P663	10.1016/S0730-725X(03)00097-3	7
14	Hwang WL, 2016, NEURO-ONCOLOGY, V18, P863	10.1093/neuonc/nov285	0

CENTRALITY

The top ranked item by centrality is Barba I, 2001, J NEUROSURG, V94, P55 in Cluster #8, with centrality of **0.58**. The second one is Cho YD, 2003, MAGN RESON IMAGING, V21, P663 in Cluster #7, with centrality of **0.53**. The third is Demir MK, 2006, EUR J RADIOL, V60, P48 in Cluster #7, with centrality of **0.52**. The 4th is Abrahams JM, 1999, J NEUROSURG, V90, P359 in Cluster #10, with centrality of **0.46**. The 5th is Galanis E, 1998, CANCER-AM CANCER SOC, V82, P1915 in Cluster #10, with centrality of **0.45**. The 6th is Preul MC, 1996, NAT MED, V2, P323 in Cluster #10, with centrality of **0.45**. The 7th is KINOSHITA Y, 1994, NEUROSURGERY, V35, P606 in Cluster #10, with centrality of **0.44**. The 8th is Ibe M, 1996, CANCER-AM CANCER SOC, V78, P133 in Cluster #10, with centrality of **0.42**. The 9th is Nakasu S, 1996, ACTA NEUROCHIR, V138, P763 in Cluster #10, with centrality of **0.42**. The 10th is SALPIETRO FM, 1994, NEUROSURGERY, V35, P638 in Cluster #4, with centrality of **0.41**.

Centrality	Node Name	DOI	Cluster ID
0.58	Barba I, 2001, J NEUROSURG, V94, P55	10.3171/jns.2001.94.1.0055	8
0.53	Cho YD, 2003, MAGN RESON IMAGING, V21, P663	10.1016/S0730-725X(03)00097-3	7
0.52	Demir MK, 2006, EUR J RADIOL, V60, P48	10.1016/j.ejrad.2006.06.002	7
0.46	Abrahams JM, 1999, J NEUROSURG, V90, P359	10.3171/jns.1999.90.2.0359	10
0.45	Galanis E, 1998, CANCER-AM CANCER SOC, V82, P1915		10
0.45	Preul MC, 1996, NAT MED, V2, P323	10.1038/nm0396-323	10
0.44	KINOSHITA Y, 1994, NEUROSURGERY, V35, P606	10.1227/00006123-199410000-00005	10
0.42	Ibe M, 1996, CANCER-AM CANCER SOC, V78, P133	10.1002/(SICI)1097-0142(19960701)78:1<133::AID-CNCR19>3.0.CO;2-0	10
0.42	Nakasu S, 1996, ACTA NEUROCHIR, V138, P763	10.1007/BF01411485	10
0.41	SALPIETRO FM, 1994, NEUROSURGERY, V35, P638	10.1227/00006123-199410000-00009	4

SIGMA

The top ranked item by sigma is Louis DN, 2016, ACTA NEUROPATHOL, V131, P803 in Cluster #0, with sigma of **6748.28**. The second one is Cho YD, 2003, MAGN RESON IMAGING, V21, P663 in Cluster #7, with sigma of **174.00**. The third is Goldbrunner R, 2016, LANCET ONCOL, V17, PE383 in Cluster #0, with sigma of **130.84**. The 4th is Barba I, 2001, J NEUROSURG, V94, P55 in Cluster #8, with sigma of **57.02**. The 5th is Nagar VA, 2008, AM J NEURORADIOL, V29, P1147 in Cluster #2, with sigma of **20.12**. The 6th is Preul MC, 1996, NAT MED, V2, P323 in Cluster #10, with sigma of **14.24**. The 7th is Deepak S, 2019, COMPUT BIOL MED, V111, P0 in Cluster #1, with sigma of **14.16**. The 8th is Demir MK, 2006, EUR J RADIOL, V60, P48 in Cluster #7, with sigma of **13.41**. The 9th is Sanverdi SE, 2012, EUR J RADIOL, V81, P2389 in Cluster #6, with sigma of **10.89**. The 10th is SALPIETRO FM, 1994, NEUROSURGERY, V35, P638 in Cluster #4, with sigma of **6.06**.

Sigma	Node Name	DOI	Cluster ID
6748.28	Louis DN, 2016, ACTA NEUROPATHOL, V131, P803	10.1007/s00401-016-1545-1	0
174.00	Cho YD, 2003, MAGN RESON IMAGING, V21, P663	10.1016/S0730-725X(03)00097-3	7
130.84	Goldbrunner R, 2016, LANCET ONCOL, V17, PE383	10.1016/S1470-2045(16)30321-7	0
57.02	Barba I, 2001, J NEUROSURG, V94, P55	10.3171/jns.2001.94.1.0055	8
20.12	Nagar VA, 2008, AM J NEURORADIOL, V29, P1147	10.3174/ajnr.A0996	2
14.24	Preul MC, 1996, NAT MED, V2, P323	10.1038/nm0396-323	10

Sigma	Node Name	DOI	Cluster ID
14.16	Deepak S, 2019, COMPUT BIOL MED, V111, P0	10.1016/j.compbiomed.2019.103345	1
13.41	Demir MK, 2006, EUR J RADIOL, V60, P48	10.1016/j.ejrad.2006.06.002	7
10.89	Sanverdi SE, 2012, EUR J RADIOL, V81, P2389	10.1016/j.ejrad.2011.06.031	6
6.06	SALPIETRO FM, 1994, NEUROSURGERY, V35, P638	10.1227/00006123-199410000-00009	4

© 2026 The Author(s). Published by Bentham Science Publisher.

This is an open access article distributed under the terms of the Creative Commons Attribution 4.0 International Public License (CC-BY 4.0), a copy of which is available at: https://creativecommons.org/licenses/by/4.0/legalcode. This license permits unrestricted use, distribution, and reproduction in any medium, provided the original author and source are credited.

HOW TO CITE:
Tang F, Wei Z, Su X, Hou L, Yang M, Zhang J. MRI and Meningioma Research: Hotspots and Trends *via* Bibliometric Analysis. Curr Med Imaging, 2026; 22: e15734056425234.
http://dx.doi.org/10.2174/0115734056425234251229184318
